# Causal identification of single-cell experimental perturbation effects with CINEMA-OT

**DOI:** 10.1038/s41592-023-02040-5

**Published:** 2023-11-02

**Authors:** Mingze Dong, Bao Wang, Jessica Wei, Antonio H. de O. Fonseca, Curtis J. Perry, Alexander Frey, Feriel Ouerghi, Ellen F. Foxman, Jeffrey J. Ishizuka, Rahul M. Dhodapkar, David van Dijk

**Affiliations:** 1 Interdepartmental Program in Computational Biology and Bioinformatics, Yale University, New Haven, CT USA; 2 Department of Pathology, Yale School of Medicine, New Haven, CT USA; 3 Department of Laboratory Medicine, Yale School of Medicine, New Haven, CT USA; 4 Department of Immunobiology, Yale School of Medicine, New Haven, CT USA; 5 Department of Internal Medicine (Oncology), Yale School of Medicine, New Haven, CT USA; 6 Interdepartmental Neuroscience Program, Yale School of Medicine, New Haven, CT USA; 7 Department of Surgery, Yale School of Medicine, New Haven, CT USA; 8 Roski Eye Institute, Keck School of Medicine, University of Southern California, Los Angeles, CA USA; 9 Department of Internal Medicine (Cardiology), Yale School of Medicine, New Haven, CT USA; 10 Department of Computer Science, Yale University, New Haven, CT USA

**Keywords:** Computational models, Software, Gene regulatory networks, Sequencing

## Abstract

Recent advancements in single-cell technologies allow characterization of experimental perturbations at single-cell resolution. While methods have been developed to analyze such experiments, the application of a strict causal framework has not yet been explored for the inference of treatment effects at the single-cell level. Here we present a causal-inference-based approach to single-cell perturbation analysis, termed CINEMA-OT (causal independent effect module attribution + optimal transport). CINEMA-OT separates confounding sources of variation from perturbation effects to obtain an optimal transport matching that reflects counterfactual cell pairs. These cell pairs represent causal perturbation responses permitting a number of novel analyses, such as individual treatment-effect analysis, response clustering, attribution analysis, and synergy analysis. We benchmark CINEMA-OT on an array of treatment-effect estimation tasks for several simulated and real datasets and show that it outperforms other single-cell perturbation analysis methods. Finally, we perform CINEMA-OT analysis of two newly generated datasets: (1) rhinovirus and cigarette-smoke-exposed airway organoids, and (2) combinatorial cytokine stimulation of immune cells. In these experiments, CINEMA-OT reveals potential mechanisms by which cigarette-smoke exposure dulls the airway antiviral response, as well as the logic that governs chemokine secretion and peripheral immune cell recruitment.

## Abstract (web summary)

CINEMA-OT is a framework that enables accurate causal inference of the effects of perturbation experiments at the single-cell level.

## Main

Cellular responses to environmental signals are a fundamental component of biological functioning, playing an integral role in both homeostasis and disease^1^. For decades, controlled perturbation experiments have been used to reveal the underlying mechanisms of biological processes. Recent advances in single-cell technologies have enabled complex experiments measuring high-dimensional phenotypes at high throughput under diverse stimulation conditions^2–8^. However, deriving biological insights from these experiments remains a challenge.

Although techniques to characterize the effects of perturbations by averaging over populations are routinely used to analyze single-cell data, methods allowing for causal single-cell perturbation analyses have not yet been explored extensively. In causal inference, the quantification of responses to perturbations is known as the treatment-effect estimation problem^9^. Throughout the text, we will borrow from the terminology of causal inference, referring to perturbations and treatments, as well as response and treatment effect, interchangeably. Ideal causal methods allow for the direct characterization of underlying confounding variation, a feature that existing single-cell analysis tools do not provide.

A great deal of variability in cellular responses to treatment may be attributable to underlying confounding variation^10^. In the case of single-cell RNA sequencing (scRNA-seq) experiments, sources of variation such as cell cycle stage, microenvironment, and pre-treatment chromatin accessibility may all act as confounding factors when performing treatment-effect estimation^11^. Collectively, confounding factors can be thought of as a cell’s underlying state that may both influence a cell’s gene expression profile, and condition treatment-induced gene signatures. Correct identification of confounders enables appropriate causal matching of cell pairs between conditions, allowing treatment-effect estimation at the single-cell level.

One well-established confounding factor that may affect treatment response is cell type. For example, widely used nucleoside-analog chemotherapeutics, such as 5-fluorouracil (5-FU), act selectively on cells in the DNA-synthesis phase of the cell cycle, killing cancer cells while minimizing effects on healthy tissue^12^. Some mutations may also drive differential response to a stimulation, as is seen with some tumors in response to transforming growth factor beta (TGF-β)^13^. Confounders may be latent or unobserved, such as different exposures of cells to a drug, which may have different effects at different concentrations within each cell.

We aim to solve this problem by introducing a causal framework permitting characterization of perturbation effects at the single-cell level. In this paper, we present causal independent effect module attribution + optimal transport (CINEMA-OT), which applies independent component analysis (ICA) and filtering on the basis of a functional dependence statistic to identify and separate confounding factors and treatment-associated factors. CINEMA-OT then applies weighted optimal transport (OT)^14–16^, a natural and mathematically rigorous framework that seeks the minimum-cost distributional matching, to achieve causal matching of individual cell pairs. The computed causal cell matching enables a multitude of novel downstream analyses, including but not limited to individual treatment-effect estimation, sub-cluster-level analysis of biological-process enrichment, treatment synergy analysis, and attribution of perturbation effects.

We demonstrate the power of CINEMA-OT by benchmarking it on several simulated and real datasets and comparing it with existing single-cell-level perturbation analysis methods. We then perform CINEMA-OT analyses of two newly generated datasets. In the first, we examine the effects of viral infection and cigarette smoke on innate immune responses in airway organoids. In the second, we perform combinatorial cytokine stimulation of ex vivo peripheral blood mononuclear cells to characterize how cytokines act in concert to shape immune responses.

### Results

#### Confounder signal matching using CINEMA-OT

To perform causal inference of perturbation effects at the single-cell level, we have adopted the potential outcome causal framework^9,17^. To generate causal assertions about the effect of a perturbation on the transcriptional state of a given cell, we ideally would measure the same cell both before and after a perturbation. However, the process of obtaining transcript measurements from single cells is destructive, and an individual cell may be measured only once. A solution is to infer counterfactual cell pairs, which are inferred causally linked pairs—predictions of what a cell in one condition would look like in another condition. The potential outcome framework formalizes this concept by establishing a rigorous statistical framework based on triplets of confounding variables, treatment and outcome variables^9,17,18^. Our task of inferring single-cell treatment effects can be translated to estimating the individual treatment effect (ITE) under the potential outcome framework^9,17,18^.

A key difficulty for applying the potential outcome framework for our task is ‘the mixing of confounders with outcomes’. In the context of causal discovery, this has also been described as learning with both interventions and latent confounding^19^. In our case, a gene can contribute to confounding variation as well as to treatment-associated variation. To apply the tools of classical causal inference, confounding factors must first be distinguished from treatment-associated factors.

**Fig. 1:**
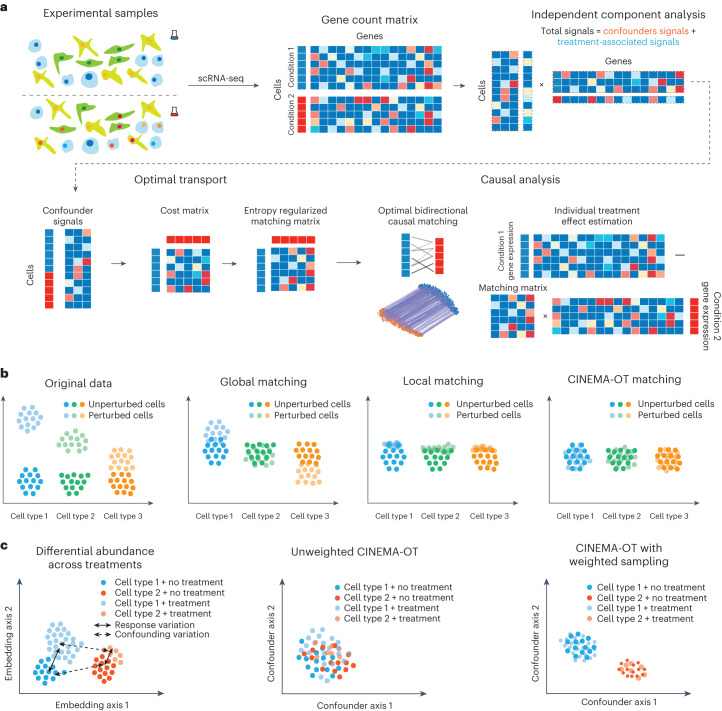
Overview of the CINEMA-OT framework. **a**, scRNA-seq count data is first decomposed into confounder variation and treatment-associated variation using ICA. Cells are then matched across treatment conditions by entropy-regularized optimal transport in the confounder space to generate a causal matching plan. The smooth matching map can then be used to estimate individual treatment effects. **b**, Illustration of the properties of CINEMA-OT compared with other potential matching schemes, including global matching (minimizing the average difference) and local matching (finding nearest neighbors for each cell). **c**, Illustration of the differential abundance issue in the unweighted CINEMA-OT method, and the resampling procedure used in CINEMA-OT-W.

To unmix confounding effects and treatment-associated effects, we propose two sufficient assumptions regarding the independence between confounding factors and treatment events, and the linearity of source signal combinations. On the basis of these assumptions, we provided the theoretical foundation that confounding factors of data obtained from ICA are identifiable if an ideal statistical test is used to analyze each component (see Supplementary Note 1). In CINEMA-OT, a Chatterjee’s coefficient-based distribution-free test is used to quantify whether each component correlates with the treatment event^20^ (Fig. 1a).

Finally, using the identified confounding factors, we apply optimal transport to generate causally matched counterfactual cell pairs. This is equivalent to applying optimal transport on the full ICA embedding while setting the treatment-associated factors to zero. Optimal transport is a natural choice for this matching procedure, because it preserves mass, is robust to outliers, and avoids collapsing matches at the boundaries of separated clusters within the data^16,21^. By contrast, global matching may have poor performance when there are confounder-specific heterogeneous responses to treatment, and local matching may be susceptible to boundary effects (Fig. 1b). While solving the optimal transport problem is often prohibitively resource-intensive for large-scale biological data, CINEMA-OT considers the tractable case of entropic regularization^15,16^. Optimal transport with entropic regularization can be formulated as a strictly convex optimization problem that can be solved efficiently using the alternating direction method (Sinkhorn–Knopp algorithm^15,16^).

There are a number of existing methods that perform perturbation-effect analysis in single-cell omics data, but none of them achieve guaranteed confounder identification, which is a necessary condition for interpretable causal-effect estimation. A thorough discussion of related methods^3–7,11,22–25,26–36^ is available in Supplementary Note 2.

#### Causal matching in the setting of differential abundance

A treatment may change the distribution of cell densities, for example cells may die or proliferate in response to a perturbation. That is, there may be differential confounder abundance across datasets of experimentally perturbed cells. Differential abundance can affect the performance of CINEMA-OT because, in this case, the underlying confounders are no longer independent of the treatment event, and our first assumption is violated. Our experiments have shown that although CINEMA-OT can tolerate moderate levels of differential abundance, it can fail when high levels of differential abundance are present (Supplementary Fig. 1).

To address the issue of differential abundance, we have developed a reweighting procedure called CINEMA-OT-W. In this procedure, before applying ICA, we first align the treated cells by their *k-*nearest neighbors (*k*-NN) in the untreated condition, similar to the perturbation signature calculation approach in Mixscape^11^. Although the resulting aligned cell populations may be imperfectly mixed, the *k*-NN alignment process groups together cells with similar confounder characteristics. We then cluster the aligned cells on the basis of the confounder space and subsample them to ensure that there is an equal ratio of treated and untreated cells in each cluster. This reweighting step effectively removes the confounding signal from the treatment event, allowing subsequent application of CINEMA-OT to successfully identify the confounders (Fig. 1c). CINEMA-OT-W greatly extends the power of the original CINEMA-OT in samples with substantial differential abundance across experimental conditions.

We note that this functionality should be used only when required. When dealing with data exhibiting differential abundance, our theoretical foundation no longer holds, meaning that the ability of any existing model, including CINEMA-OT-W, to identify certain classes of cellular responses accurately may be reduced. Additionally, selecting the optimal resolution of clustering in CINEMA-OT-W may require prior biological knowledge, because suboptimal choices of clustering resolution could result in reduced power to identify distinct cell populations. As an alternative to CINEMA-OT-W, CINEMA-OT also provides an option to assign weights according to user-provided labels (for example cell types). In this case, CINEMA-OT can sample data using confounder labels instead of automatically balancing over all possible covariates.

#### Causal matching enables various downstream analyses

**Fig. 2:**
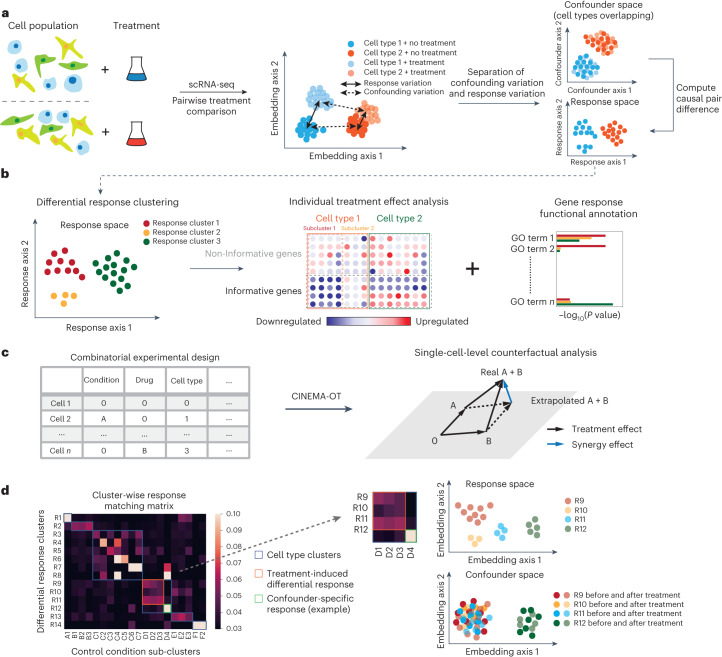
Key functionalities of CINEMA-OT. **a**, CINEMA-OT takes scRNA-seq data labeled by treatment condition as input. CINEMA-OT learns a confounder embedding that is mixed across batches and matches counterfactual cell pairs across conditions to compute causal perturbation effects. **b**, The single-cell-level treatment-effect matrices can be further clustered, and gene set enrichment analysis can be conducted on the output. GO, Gene Ontology. **c**, Single-cell-level synergy in combinatorial perturbations can be obtained as the dissimilarity of extrapolated phenotypes and true combinatorially perturbed phenotypes. **d**, CINEMA-OT can attribute divergent treatment effects to either explicit confounders or latent confounders by analysis of cluster-wise response matching matrices.

The matched counterfactual cell pairs computed by CINEMA-OT define two key outputs: (1) the matching correspondence matrix across treatment conditions, and (2) the individual treatment effect (ITE) for each cell with its counterfactual pair across treatments (Fig. 2a).

Individual treatment-effect (ITE) matrices are cell by gene matrices that can be clustered and visualized by existing scRNA-seq computational pipelines. By clustering over an ITE matrix, we can identify groups of cells with a shared treatment response. We can then perform a statistical analysis to identify the genes with significant response magnitudes in each group and identify their coordinated biological function by gene set enrichment analysis (Fig. 2b).

In addition, when experimental data are available for multiple treatments performed in combination (for example, control, treatment A, treatment B, and combined treatment A+B), we can define a synergy-effect metric by comparing the predicted effect of combining multiple treatments with the observed effect of combined treatment (Fig. 2c). We define this synergy metric by estimating the difference between the true sample under combined treatment (A+B) and the predicted sample by adding the effects of treatment A and treatment B, thus assuming the effects are purely linear and non-interactive. If no difference is measured, we may conclude that there are no nonlinear or interaction effects between the treatments. If non-zero synergy is present, this points to some interaction between treatments A and B. Synergy is computed for every cell–gene pair, resulting in a matrix of equivalent form to the expression and ITE matrices—a unique feature of CINEMA-OT. Notably, as the synergy serves as a summary statistic of the combinatorial cellular responses, the same synergy value may correspond to a number of underlying mechanisms. For instance, for gene *x*, synergistic activation (*x*_A+B_ > *x*_A_ = *x*_B_ = *x*_control_ = 0) and uniform inhibition (*x*_control_ > *x*_A+B_ ≈ *x*_A_ ≈ *x*_B_) may lead to the same level of positive synergy. Our synergy metric enables unbiased investigations of nonlinear treatment effects.

Another important task in perturbation-effect analysis is the attribution of treatment effects. Differential response can be driven either by differences in explicit confounding factors or by latent factors, such as treatment heterogeneity. Because CINEMA-OT provides a single-cell-level matching as one output, the task can be solved by analysis on the clustered matching matrix. Responses that cluster both in response and in confounder space may be attributed to explicit confounding factors. Conversely, responses that cluster well in the response space but do not demonstrate clustering in the confounder space may be attributed to latent factors (Fig. 2d). Such an analysis can be performed either at the cell-type level or at the sub-cluster level to reveal underlying heterogeneity. To further identify genes with explicit confounder-specific treatment effects, we quantify the confounder-effect size via a causal regression model and estimate its relative strength using the ratio of confounder-explained effect size to the residual norm (see Methods for additional details).

#### Validation of CINEMA-OT using simulated data

To investigate how CINEMA-OT differs from existing single-cell-level methods for perturbation-effect analysis in practice, we first perform extensive benchmarking on a number of tasks in simulated scRNA-seq data. Our study involves a meticulous comparison of existing methods, including a method we refer to as Mixscape that calculates the perturbation signature^11^ and is considered in CoCoA-diff^37^, scGen^22^, CPA^23^, ContrastiveVI^24^, and CellOT^25^, with two variants of our approach with or without sampling (CINEMA-OT-W, CINEMA-OT). Moreover, we explore the potential benefits of integrating batch-effect analysis into Mixscape analysis, a method we refer to as Harmony-Mixscape^38^. Additionally, we include a direct optimal transport (Full OT), applied on the original data (without separation of treatment-associated and confounding factors) as an ablation study showing the essence of modeling confounding variation in our approach. Our comparison is based on three categories of metrics:

1. Cell distribution equalization after treatment-effect removal. In datasets with or without ground truth, we can measure the validity of treatment effects by examining cell distributions in the gene expression space after removal of treatment effects. If different treatments are applied to the same confounder distribution, then these distributions should overlap well after treatment effects are removed. Metrics for evaluating treatment-effect removal include average silhouette width and principal components regression score (PCR).
2. Differential response cluster preservation. If a cell population has divergent responses to a perturbation, the cell population would form clustering structures in the response space. Therefore, preservation of such clustering structures in the estimated treatment effects is essential for identification of perturbation effects. In this study, we evaluate the cluster preservation level using an adjusted Rand index in ITE matrices.
3. Attribution accuracy. Differential response patterns can be attributed to either confounder-specific effects (for example cell-type-specific effects) or latent-factor-driven effects (for example treatment drug dose distribution). In simulated data, the attribution accuracy can be measured through independence between confounding factors and responses conditioned on ground-truth response labels. In our study, this is evaluated by the PCR in ITE matrices.

**Fig. 3:**
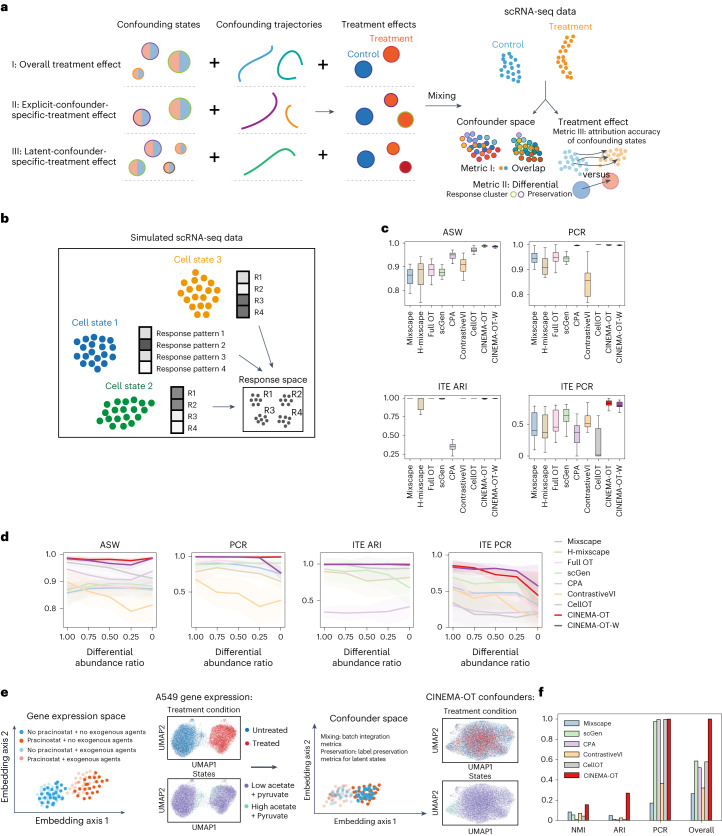
Benchmarking of CINEMA-OT against other methods for single-cell perturbation analysis. **a**, Illustrations of the data stimulation and metrics included in the benchmarking. **b**, Illustration of our conducted simulation study. **c**, Box plots of different validation metrics on synthetic data for CINEMA-OT and other methods (*n* = 15 for confounder embedding metrics, *n* = 12 for ITE metrics). ITE metrics were computed only for datasets with differential responses. The top and bottom hinges represent the top and bottom quartiles, and whiskers extend from the hinge to the largest or smallest value no further than 1.5 × the interquartile range from the hinge. The median is used as the center. ASW, Average silhouette width. ARI, adjusted Rand index. **d**, Comparison of the performance of different methods across synthetic datasets with various differential-abundance ratio settings: 1, 0.75, 0.5, 0.25, 0 (missing cell types), with *n* = 15 for confounder embedding metrics and *n* = 12 for ITE metrics in each setting. Data are presented as mean values ± s.d. **e**, Illustration of the validation of CINEMA-OT on the Sciplex dataset. **f**, Quantification of different validation metrics on the Sciplex dataset for CINEMA-OT and other methods.

We considered the dependence between confounders and ground-truth treatment effects in three settings: (1) overall treatment-effect modeling of common responses, regardless of confounders; (2) confounder-specific treatment-effect modeling of diverging responses driven by underlying confounders, such as cell-type-specific response; and (3) latent-factor-driven treatment-effect modeling of the differential treatment effect caused by unobservable latent confounders. The genes in each setting are separated into three subsets, corresponding to the underlying trajectory, cell types, and treatment-associated genes, respectively (Fig. 3a). In our simulated data, all settings were covered together by modeling the differential response probabilities as conditional distributions on confounder clusters (Fig. 3b). Additionally, we examine the impact of differential abundance on the performance of various methods by selectively subsampling cells from half of the confounder clusters in the treated condition. We refer to this subsampling ratio as the differential abundance ratio (DA ratio) in the following sections. Furthermore, we have investigated the relationship between the performance of single-cell-level treatment analysis and the signal-to-noise ratio of an scRNA-seq dataset by downsampling the gene counts of simulated datasets at different levels.

Before our evaluations, the optimal hyperparameter setting for each method was selected through parameter-sweep analysis (Methods and Supplementary Fig. 2). Our quantitative assessment of these synthetic datasets shows that, in the case of balanced confounder states (no differential abundance), CINEMA-OT (or CINEMA-OT-W) achieves the best performance among all tested methods in batch mixing and treatment-effect attribution, while most methods, including CINEMA-OT (and CINEMA-OT-W), succeed in differential-response cluster preservation (Fig. 3c).

**Extended Data Fig. 1:**
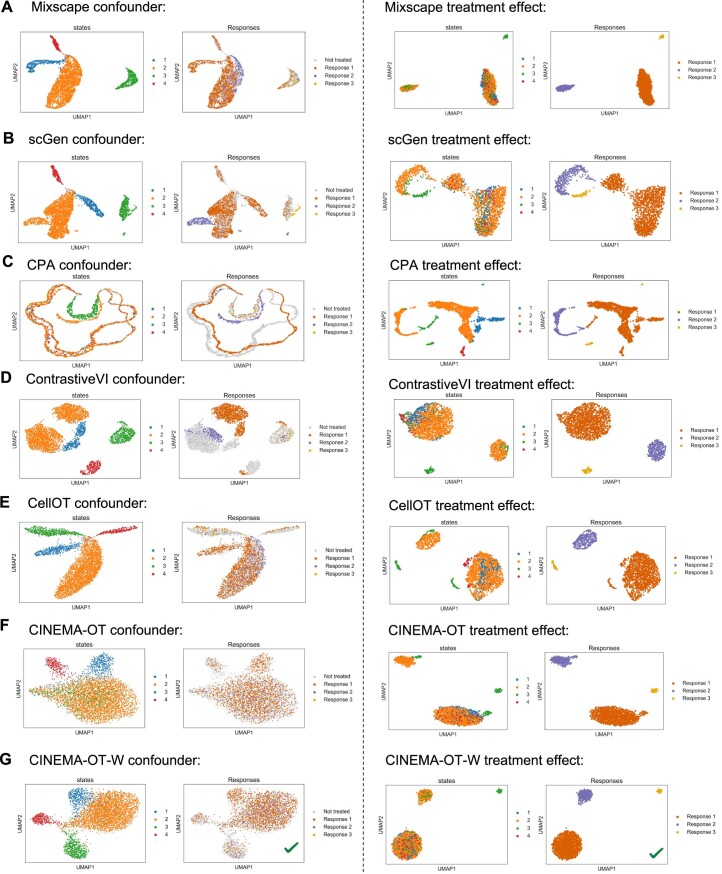
CINEMA-OT validation on a simulated dataset example with differential abundance. A-G. UMAP visualizations of confounder space (left) and treatment effect space (right), both colored by cell type ("states") and response patterns ("Responses") in a simulated dataset with differential abundance ratio = 0.25.

**Extended Data Fig. 2:**
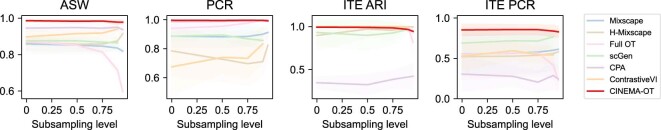
Comparison for different single-cell level treatment effect analysis methods’ performance as the sparsity level of the dataset increases. Here the result of scGen and ContrastiveVI in the setting of subsampling level = 0.95 is not shown, as they fail to converge or return NaN in some datasets. Data are presented as mean values +/- std (n=15 for confounder embedding metrics, n=12 for ITE metrics). The smaller number of n for ITE metrics is because they are only meaningful for datasets with response pattern numbers larger than 1.

By varying the differential-abundance level, we have found that the original version of CINEMA-OT performs better than CINEMA-OT-W does when the differential-abundance level is small (DA ratio ≥ 0.75), but CINEMA-OT-W performs better in treatment-effect attribution at a higher differential-abundance level (DA ratio ≤ 0.5). In both cases, CINEMA-OT substantially outperforms other methods in treatment-effect attribution, while performing as well as other methods in cell-distribution equalization and preservation of differential-response clusters (Fig. 3d). The superior performance of CINEMA-OT-W in the datasets with substantial differential abundance is also shown by qualitative visualizations of both confounder space (where the response cluster should be mixed and the cell states should be distinctive) and the treatment-effect space (where the response cluster should be distinctive while the cell states should be mixed) (Extended Data Fig. 1). Through our experiments with varying levels of data sparsity, we have found that, even under a high sparsity level, CINEMA-OT’s performance decreases only slightly with increasing sparsity, maintaining its lead in accurately attributing responses while achieving top performance in preserving batch mixing and differential response clusters (Extended Data Fig. 2).

**Extended Data Fig. 3:**
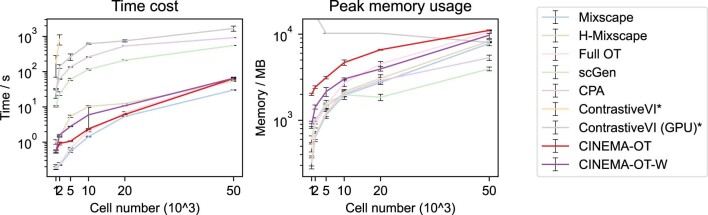
Running time and peak memory usage comparison for different single-cell level treatment effect analysis methods. *: These methods are tested on high performance clusters, therefore the performance may not be directly comparable. Data are presented as mean values +/- std. The sample size equals 5 in each setting with the exception of scGen, CPA, and ContrastiveVI(GPU) with n_cells_ = 50000, in which settings the sample size is set to 2 to save time.

Finally, we performed a benchmarking study of run time and peak memory usage on a series of subsampled scRNA-seq data, containing 1,000, 2,000, 5,000, 10,000, 20,000, or 50,000 cells. Our results show that CINEMA-OT and CINEMA-OT-W perform nearly as quickly as the fastest method available (Mixscape), markedly outperforming deep-learning-based approaches in speed, with a run time of approximately 1 min for 50,000 cells (Extended Data Fig. 3). Although the peak memory usage of CINEMA-OT (and CINEMA-OT-W) is substantial owing to the use of a dense matching matrix across conditions, it still requires less than 12 GB of memory for 50,000 cells, making it possible to run on most modern laptop computers (Extended Data Fig. 3). We also implemented an experimental version of CINEMA-OT that allows handling larger datasets by adopting advanced OT solvers in the ott-jax library^39^.

#### Validation of CINEMA-OT using real data

To evaluate the performance of CINEMA-OT in a real setting, we used two publicly available single-cell transcriptomics datasets: (1) sequencing of entorhinal cortex in people with Alzheimer’s disease and unaffected controls^40^; and (2) the sci-Plex4 drug perturbation dataset^8^, which measures the response of the A549 and MCF7 cell lines to perturbation with 17 drugs.

**Extended Data Fig. 4:**
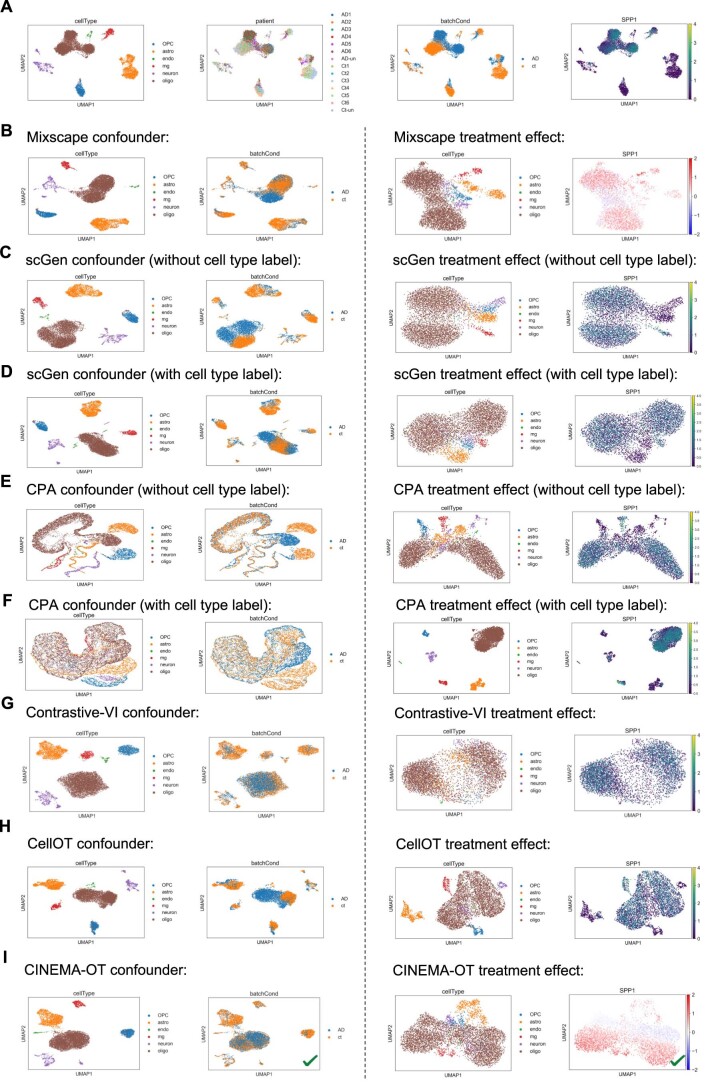
CINEMA-OT validation on Alzheimer’s data. A. UMAP visualizations of different covariates (Cell type, donor batch, and AD/control) and SPP1 expression. B-I. Different methods’ confounder space visualization and treatment effect visualization, colored by cell type and condition (for confounder space), cell type and SPP1 expression (for response space) respectively.

**Extended Data Fig. 5:**
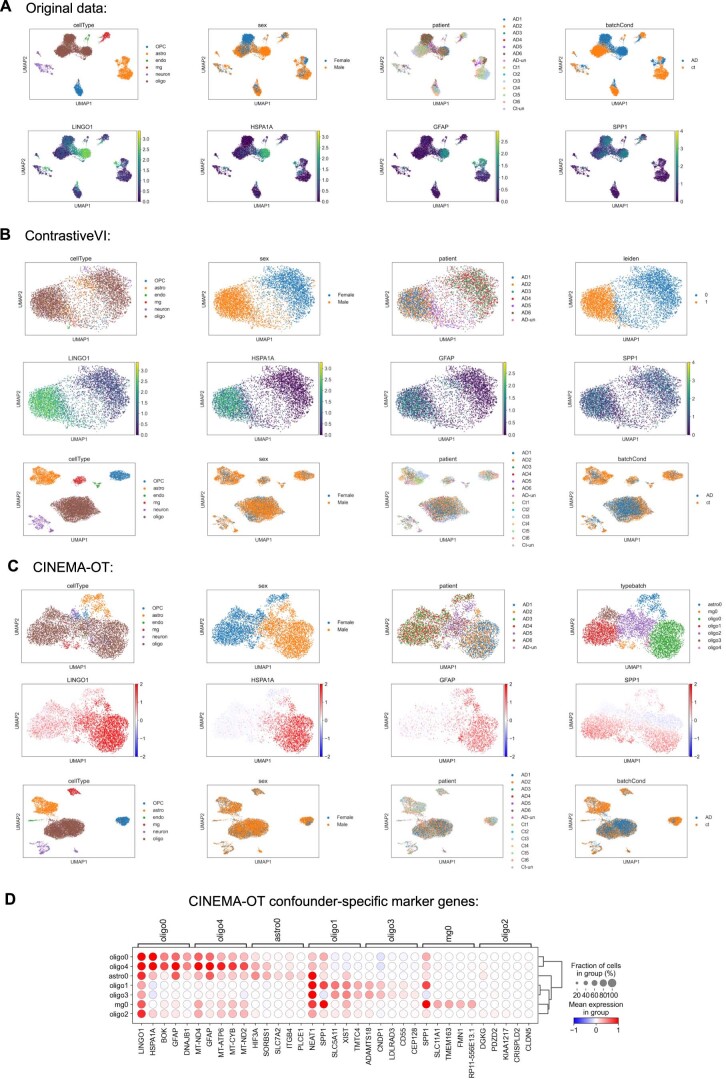
Additional comparisons of ContrastiveVI and CINEMA-OT in the Alzheimer scRNA-seq dataset. A. UMAP visualizations of different covariates (Cell type, donor sex, donor batch, and AD/control) and expressions of different cell-type specific AD marker genes. B. ContrastiveVI response space visualization for covariates and leiden subclusters (first line), state-specific gene visualization in the response space (second line), and confounder space visualization for covariates (third line). C. CINEMA-OT response space visualization for covariates and Leiden subclusters (first line), state-specific gene visualization in the response space (second line), and confounder space visualization for covariates (third line). D. Response-cluster specific genes identified by CINEMA-OT.

In the Alzheimer’s disease dataset, we focused on qualitative comparison of perturbation-effect removal and differential response cluster preservation. While the first comparison can be conducted in an unsupervised manner, for the second comparison, we integrated prior knowledge to evaluate the preservation of clusters of interest^41^. One notable example gene is *SPP1*, which has been described as being upregulated in some cell types of people with Alzheimer’s disease (for example microglia and some neuronal subtypes), but not in others (for example endothelial cells)^40,42^. We compared CINEMA-OT with Mixscape, scGen, CPA, ContrastiveVI, and CellOT in our experiments, covering both the default model (cell-type-unaware) and cell-type-aware models for scGen and CPA. The visualizations of confounding spaces and treatment effects identified by each method can be seen in Extended Data Fig. 4. Our results show that the other methods, in general, either preserve the differential response of SPP1 by automatic clustering (Mixscape, scGen, CPA without cell-type label) or mix cell distributions well in the latent space (ContrastiveVI, CellOT), but not both. By contrast, CINEMA-OT succeeds in both tasks (Extended Data Figs. 4 and 5).

**Extended Data Fig. 6:**
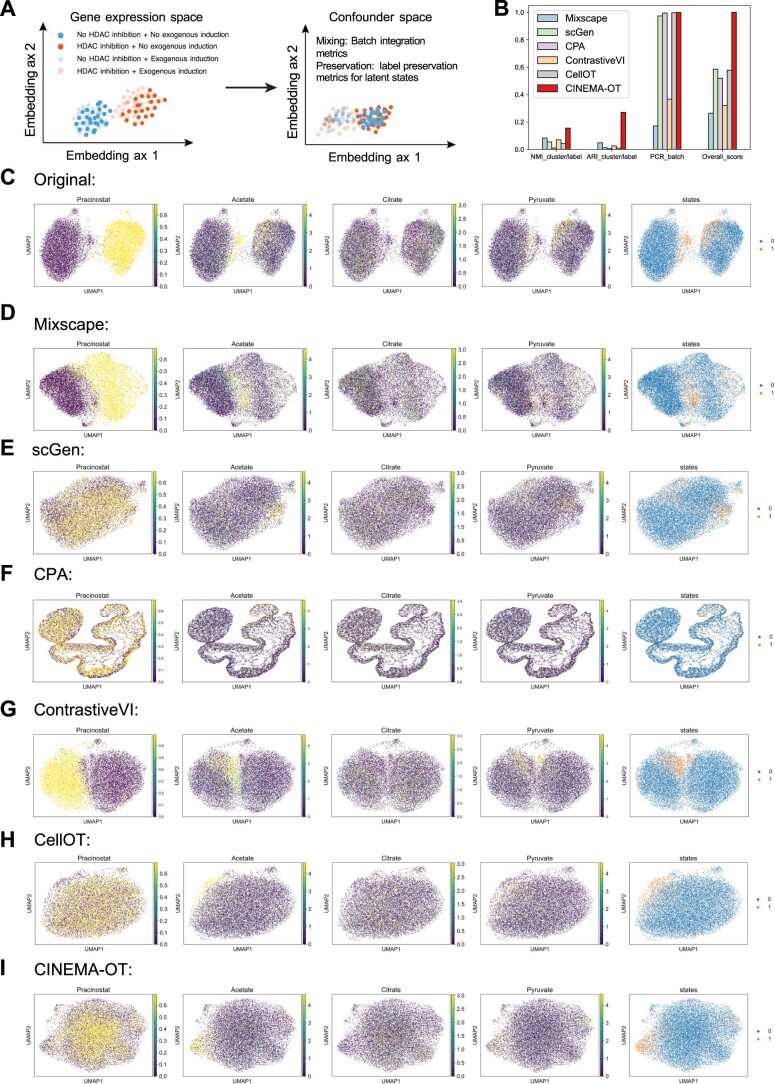
CINEMA-OT validation on Sciplex data. A. Schematic overview of the benchmarking design, same as the overview shown in Fig. 3e. B. The bar plot showing metric comparison results across benchmarked methods, same as Fig. 3f. C-I. UMAP visualization of different methods’ confounder space, colored by the perturbation level of Pracinostat, Acetate, Citrate, Pyruvate, and the defined confounder states respectively.

In the Sciplex dataset, we investigated the response to perturbation with pracinostat (SB-939), a histone deacetylase (HDAC) inhibitor, with the combinatorial induction of exogenous acetate, citrate, and pyruvate. HDAC inhibitors act as antitumoral agents by antagonizing the pro-transcriptional effects of histone deacetylation and silencing the expression of oncogenic factors through chromatin remodeling^43^. As HDAC inhibitors act partly through the deprivation of acetyl-CoA, we expect that the relative abundance of acetyl-CoA precursors within a cell would modulate the effect of HDAC inhibitor exposure, and acetyl-CoA precursors can be considered confounders^8^ (Fig. 3e). Indeed, in the uniform manifold approximation and projection (UMAP) embedding of the A549 cell line across two doses of SB-939, within each dose population, the cell neighborhood relationship is determined by doses of exogenous acetate, citrate, and pyruvate, separating the entire cell population into two latent-confounder states. Ideally, a treatment-effect analysis method should not only achieve good mixing in the confounder space, but also automatically match the cells by the latent states to accurately specify the treatment effect. The two aspects can be quantitatively validated for each method by employing batch-mixing metrics and label-preservation metrics in the confounding space. Among all tested methods, CINEMA-OT achieves superior performance in both aspects, as suggested by our qualitative and quantitative evaluations (Fig. 3f and Extended Data Fig. 6).

#### CINEMA-OT identifies synergy of smoke and virus infection

**Fig. 4:**
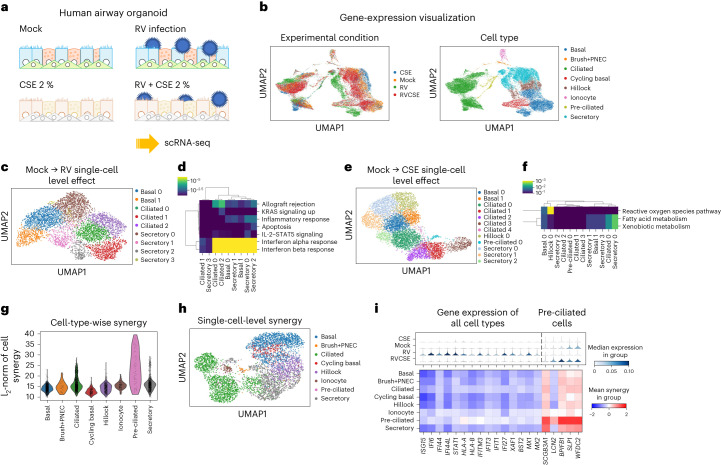
CINEMA-OT identifies a heterogeneous defensive response in human airway epithelial cells exposed to rhinovirus and cigarette-smoke extract. **a**, Overview of experimental design. Differentiated airway epithelial organoids are challenged with mock (control) or RV infection, with or without CSE exposure. **b**, UMAP projection of expression data labeled by perturbation and cell type. **c**, UMAP projection of the individual treatment-effect matrix obtained by CINEMA-OT from the RV response without CSE exposure, colored by response cluster. **d**, Gene set enrichment analysis of mock to RV response clusters identified by CINEMA-OT, colored by adjusted *P* value. **e**, UMAP projection of the individual treatment effect matrix obtained by CINEMA-OT from the CSE response without RV exposure, colored by response cluster. **f**, Gene set enrichment analysis of mock to CSE response clusters identified by CINEMA-OT, colored by adjusted *P* value. **g**, Cell-wise synergy score visualization. **h**, UMAP visualization of CINEMA-OT synergy embedding, colored by cell type. **f**, Two diverging patterns among CINEMA-OT identified synergistic genes, visualized by stacked violin plots and dot plots.

In addition to benchmarking CINEMA-OT against other methods, we have applied CINEMA-OT to new scRNA-seq data about rhinovirus infection in primary human bronchial organoids (Fig. 4a). The experiment comprises four conditions: exposure to cigarette-smoke extract (CSE), rhinovirus (RV) infection, the combination of rhinovirus and cigarette smoke (RVCSE), and a control condition (mock). Although rhinovirus infection has been investigated^44^, the goal of our study was to probe cellular defense responses to viral infection from each airway epithelial cell type in the presence or absence of a common environmental insult that is known to impact the outcome of rhinovirus infection: cigarette smoke. Previous studies of viral infection using this model considered gene expression in each cell type, but not heterogeneous response patterns, which may be of biological and clinical relevance in understanding the tissue response to respiratory virus infections^44,45^.

We first performed preprocessing of the dataset and annotated eight cell clusters in total, including major cell types in the airway (basal, secretory, ciliated) and other rare (ionocyte, pulmonary neuroendocrine cells (PNECs), and brush) or transitional (hillock and pre-ciliated) cell types (Fig. 4b). We then performed CINEMA-OT analysis on mock–RV and mock–CSE condition pairs to identify single-cell-level treatment effects (the ITE matrix). For both condition pairs, CINEMA-OT returns batch mixed confounder embedding and reasonable response clusters (Supplementary Figs. 4 and 5). As expected, in response to RV infection, most epithelial cell types exhibited robust induction of the interferon response, with upregulation of several interferon-stimulated genes, including *ISG15*, *IFI44*, *STAT1*, *MX1*, and others (Fig. 4c,d)^44^. In response to CSE exposure, a subset of epithelial cells increased expression of genes associated with metabolism of reactive oxygen species (*PRDX1*, *TXN*) as well as genes related to fatty acid and xenobiotic metabolism (*ADH1C*, *ALDH3A1*, *CYP1B1*). Interestingly, responses to CSE were primarily enriched in particular cell subpopulations, including hillock, ciliated, and secretory cells, in contrast to the global interferon response that was seen following rhinovirus infection (Fig. 4e,f). This demonstrates a functional division of defense mechanisms in the airway epithelium, with cell-type-specific responses to different insults.

After analysis of the effect of cigarette smoke and viral infection individually, positive and negative synergy between these two insults was assessed by calculating cell–gene synergy scores (Fig. 4g,h). We found that, among the strongest synergistic effects, interferon-stimulated genes (ISGs) exhibited negative synergy in general when cells were exposed to RV and CSE. ISGs showed a global reduction during viral infection in the presence of cigarette smoke compared with viral infection alone (Fig. 4i), consistent with previous mechanistic studies showing that the antioxidant defense response induced by CSE suppresses signaling pathways required for induction of interferon-stimulated genes in response to viral RNA in airway epithelial stem cells^46^.

In addition to a global attenuation of the interferon response, we discovered that pre-ciliated cells, in particular, exhibit pronounced synergistic expression of a distinct set of genes when co-exposed to RV and CSE (Fig. 4g,i). Pre-ciliated cells, sometimes referred to as ‘deuterosomal’ cells, are developing multiciliated cells with the marker genes *CCNO* and *CDC20B*^47^. Pre-ciliated cells co-exposed to both viral infection and CSE show synergistic induction of genes encoding secreted proteins that are typically associated with secretory cells in resting cultures, including *SCGB3A1*, *LCN2*, *BPIFB1*, *SLPI*, and *WFDC2* (Fig. 4i). This pattern could arise from pre-ciliated cells adopting a more secretory phenotype during co-exposure, or secretory cells adopting a pre-ciliated phenotype. These findings highlight the use of CINEMA-OT to identify synergistic effects on gene expression induced by co-exposure to viral infection and cigarette smoke.

#### CINEMA-OT reveals principles of innate immunity modulation

**Fig. 5:**
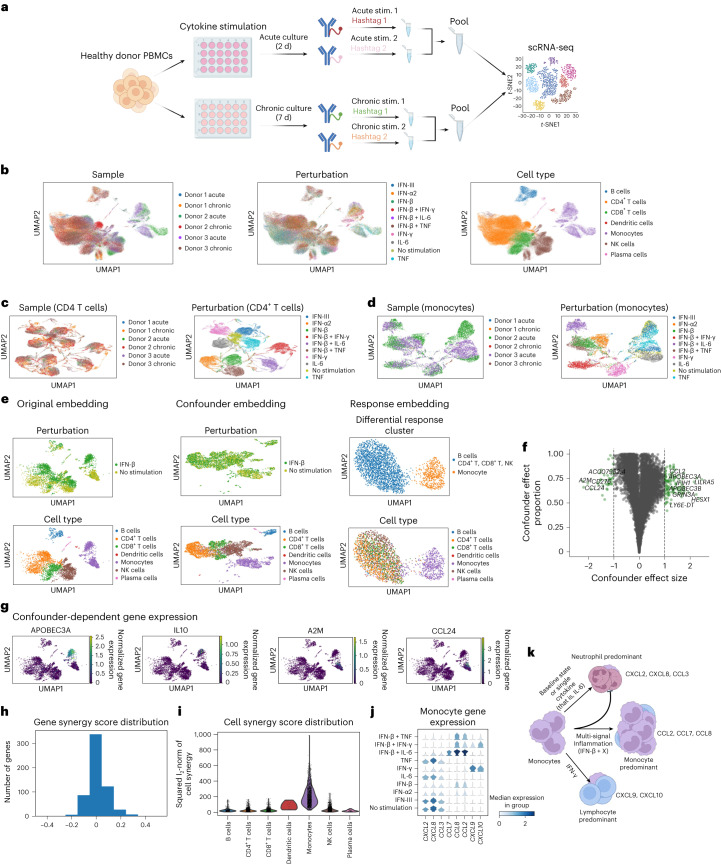
CINEMA-OT reveals combinatorial mechanisms of acute and chronic cytokine stimulation. **a**, Illustration of experimental design. **b**, UMAP projection of expression data colored by samples, perturbations, and cell types. In sample labels, H refers to the donor number, and D refers to the number of days of stimulation. NK, natural killer. **c**, UMAP projection of the CD4^+^ T cell counterfactual space from CINEMA-OT. Projections are colored by experimental batch and perturbation type. **d**, UMAP projection of the monocyte counterfactual space from CINEMA-OT. Projections are colored by experimental batch and perturbation type. **e**, UMAP projections of the original data, confounder embedding, and individual treatment effects identified by CINEMA-OT after acute stimulation with IFN-β in H3D2, colored by response cluster and cell type. **f**, Volcano plot highlighting genes with strong confounder-specific treatment effects. **g**, Normalized expression of representative confounder-specific treatment-associated genes in original UMAP space. **h**, Distribution of gene synergy score, obtained by combining results of IFN-β and TNF, IFN-β and IFN-γ, and IFN-β and IL-6 treatments in H3D2. **i**, Cell-wise synergy score visualization in the acute condition, taking a single experimental batch (H3D2) as an example. **j**, Stacked gene expression violin plot of synergistic chemokines identified by CINEMA-OT. **k**, Patterns of chemokine secretion programmed by single or multi-signal cytokine stimulation. X: TNF, IFN-γ and IL-6.

Type I, type II, and type III interferons (IFNs) act as central regulators of immune responses during intracellular pathogen infection, cancer, and in auto-immunity. However, despite the identification and adoption within the literature of a core set of interferon-stimulated genes (ISGs), IFN responses can vary widely by cell type, by individual, by IFN stimulus type, by chronicity of exposure, and by combination with signals delivered by other cytokines. In other words, the interferon response is highly context dependent. This complexity, heterogeneity, and context-specificity of IFN signaling can lead to counterintuitive results. For example, IFN-γ has been proposed to play both stimulatory and suppressive roles in cancer, and type I IFNs are used both as an immunosuppressant to treat multiple sclerosis and as immunostimulatory adjuvant treatments for cancer (for example melanoma) and chronic viral infection (for example HCV)^48–50^. To characterize the complexity of IFN signaling, we subjected peripheral blood mononuclear cells (PBMCs) from multiple healthy donors to acute (2 d) or chronic (7 d) stimulation with type I, type II, and type III IFNs, separately as well as in combination with other cytokines, such as tumor necrosis factor (TNF) and interleukin-6 (IL-6) (Fig. 5a,b).

To understand the underlying structure of the cellular response of PBMCs to interferon stimulation, we used CINEMA-OT to match treatment conditions to the untreated (control) condition. This analysis highlights the underlying hierarchical structure of cellular responses. As the hierarchical structure of cytokine response can vary with cell type, besides the regular CINEMA-OT analysis based on a single patient condition (Supplementary Fig. 5), we pooled CD4^+^ T cells and monocytes across individuals and experimental batches and performed CINEMA-OT analysis. In this case, a confounder is defined by each different experimental batch. In CD4^+^ T cells, the response can be characterized by four meta-perturbation clusters: no stimulation, IFN-γ, IL-6, TNF; IFN-α2, IFN-β, IFN-β and TNF; IFN-β and IL-6; and IFN-β and IFN-γ (Fig. 5c). In monocytes, a similar structure is observed, except that IFN-γ in monocytes represents a distinct response cluster in the phenotypic space (Fig. 5d).

**Extended Data Fig. 7:**
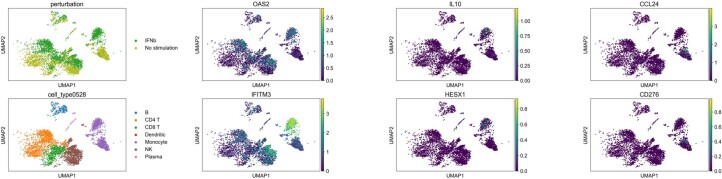
Visualizations of identified genes with strong confounder-dependent treatment effect, following Fig. 5f. UMAP visualizations of identified genes with strong confounder-dependent treatment effect, colored by perturbation condition, cell type annotation, and log normalized gene expression.

**Extended Data Fig. 8:**
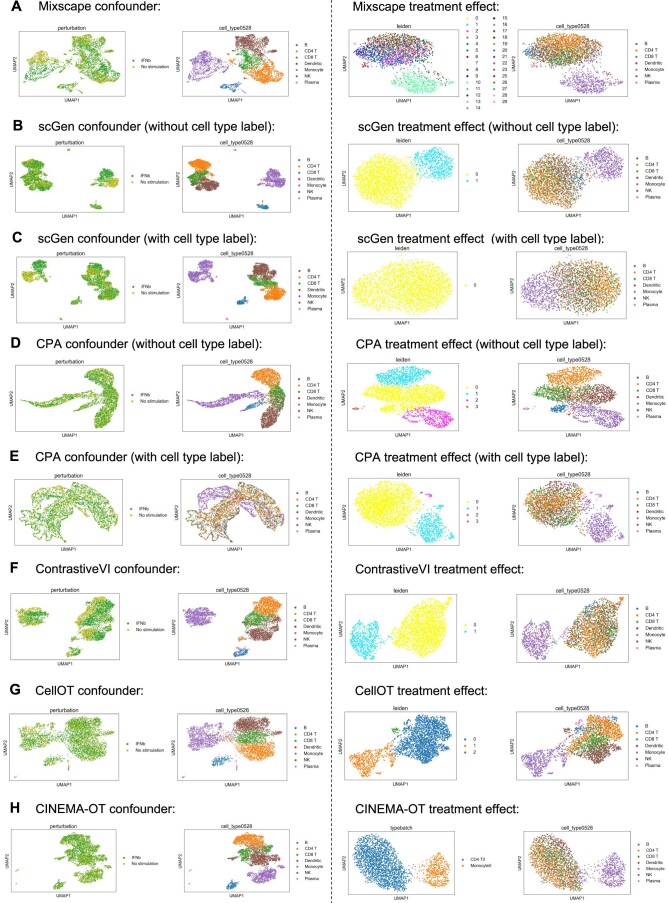
CINEMA-OT validation on the interferon data. A-H. Different methods’ confounder space visualization (colored by treatment condition and cell types) and treatment effect visualization (colored by cell types and Leiden clusters of the response space).

**Extended Data Fig. 9:**
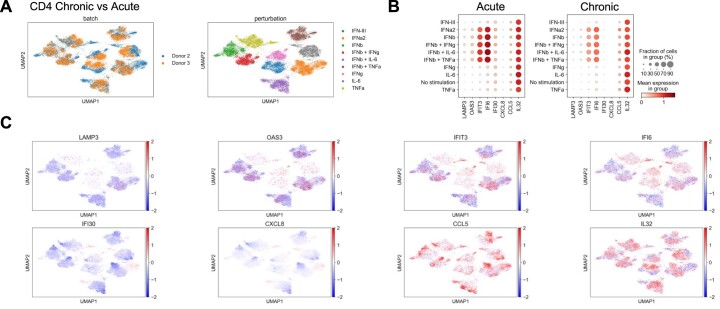
Comparison of CD4 T cells gene expression change between chronic stimulation condition and acute stimulation condition across different conditions. A. UMAP visualizations of CD4 T cell response space pooled over different perturbations, colored by donor and perturbation condition. B. Dot plots of selected genes identified by CINEMA-OT. C. Single-cell expression differences of selected genes visualized in the UMAP space.

Next, to demonstrate CINEMA-OT’s power in general single-cell-level treatment analysis, we focused on analyzing the treatment effects of IFN-β in a single experimental batch (H3D2). CINEMA-OT analysis highlights the induction of coordinated immune responses across cell types along with cell-type-specific responses, as shown in the confounder-specific effect volcano plot. For example, despite a global change in interferon-stimulated genes (Extended Data Fig. 7), monocytes demonstrate a unique program characterized by increased *APOBEC3A* and *IL10* expression and decreased *A2M* and *CCL24* expression compared with other cell types (Fig. 5e–g and Extended Data Fig. 7). Notably, a similar qualitative analysis that we performed on the Alzheimer’s dataset shows that CINEMA-OT achieves both good batch mixing and reasonable response clustering, compared with alternative methods that we tested (Extended Data Fig. 8). CINEMA-OT was also used to investigate the treatment effects of chronic versus acute stimulation in CD4^+^ T cells and reveals the attenuation of genes involved in the core type I IFN response (Extended Data Fig. 9).

To estimate the synergistic effects of acute combinatorial cytokine stimulation, we used CINEMA-OT to calculate cell–gene synergy scores. We next performed gene synergy score analysis by computing the gene-wise synergy score (Methods). The gene synergy score analysis identified genes that were synergistically induced by each combinatorial perturbation (Fig. 5h). On the basis of selected synergy genes, we summarized the cell-wise synergy effect by taking the norm over selected synergy genes. We have found that monocytes exhibit the strongest synergistic regulation compared with other cell types (Fig. 5i). Further enrichment analysis identified a number of chemokines with specific synergistic expression in monocytes with respect to different interferon perturbation (Fig. 5j and Supplementary Fig. 6). These chemokines exhibit synergistic patterns of response to multi-signal inflammation (for example IFN-β and IL-6) in monocytes, including inhibition of baseline neutrophil chemotactic signaling and induction of a monocyte chemotactic signaling program. The addition of IFN-γ contributes lymphocyte-predominant chemokines while maintaining core inflammatory programming (Fig. 5j,k). These results suggest that CINEMA-OT, when applied to combinatorial experiments, is capable of revealing the synergistic logic governing cellular signaling in inflammation and tissue repair.

### Discussion

With rapidly developing high-throughput screening technologies and an ever-rising number of datasets, single-cell-level analysis of experimental effects is becoming a critically important task in biological discovery. Current analytical approaches aiming to address this need face a number of challenges. When treatment effects are confounder specific and do not change relative cell proportions, differential-abundance methods may be unsuitable for extracting the dependence between confounder states and treatment responses. Recent neural-network-based methods for characterizing perturbation effects learn nonlinear interactions between confounders and treatment effects, but these can be prone to overfitting and have limited interpretability. In response to these challenges, we present CINEMA-OT, a framework for single-cell causal treatment-effect analysis. By explicitly separating confounder and treatment signals and matching at the single-cell level, CINEMA-OT produces a per-cell view into the effects of experimental perturbations and conditions including disease states.

We applied CINEMA-OT in several use cases, including synthetic and real datasets. In benchmarks, CINEMA-OT was able to outperform other methods in experimental-perturbation analysis. In human airway organoids, CINEMA-OT revealed how CSE can interfere with the normal innate immune response to RV infection. In combinatorial cytokine stimulation of ex vivo human peripheral immune cells, CINEMA-OT revealed complex logic that may underlie the specific recruitment of cells from the periphery to tissues responding to various injuries.

Two potential challenges for CINEMA-OT can arise owing to bias–variance trade-offs in optimal transport and the magnitude of batch effect versus biological-perturbation effect. For the first challenge, a large smoothness threshold in the entropy-regularized OT method can overly smooth the obtained matching and cause false positives by incorrectly identifying confounder variation as treatment-associated variation. However, too small a threshold would both harm the method’s stability and cause high variance. In practice, as CINEMA-OT is highly scalable, an adequate threshold can be chosen on the basis of repeated runs with different parameter settings. For the second challenge, as CINEMA-OT performs matching in the confounder space, the confounding space identified by CINEMA-OT and the optimal transport matching plan are minimally altered by the level of batch effect, as the batch effect can be viewed as a treatment-induced factor itself. However, because the current implementation of CINEMA-OT does not perform count modeling, the differential expression analysis at the gene level may be still affected by the batch effect when it causes substantial distortions of global gene expression. In this case, the confounder embedding and the matching scheme identified by CINEMA-OT can still serve as a basis for conducting advanced differential expression testing approaches, such as MiloDE^51^.

CINEMA-OT is designed to estimate causal treatment effects from experimentally perturbed single-cell omics measurements. CINEMA-OT is not able to extrapolate, meaning it cannot identify the causal effect of unmeasured perturbation-cell pairs. Integrating prior knowledge (such as ChemCPA^52^ and expiMap^53^) to achieve causally meaningful extrapolation for unseen perturbation effects remains a promising future direction. Moreover, although we have implemented a reweighting procedure to account for differential confounder abundance that may arise in response to treatment, CINEMA-OT is not designed for cases in which changes to confounder distributions are the primary effects of interest. In those cases, tools such as MELD, MILO, or DA-seq may be more suitable^54–56^.

We anticipate that, as a highly explainable and scalable causal framework, CINEMA-OT will be widely adopted for single-cell perturbation analysis.

## Methods

### CINEMA-OT

CINEMA-OT is an unsupervised method for separating confounding signals from perturbation signals for matching cells through imputing counterfactuals and computing perturbation effect at a single-cell level (https://github.com/vandijklab/CINEMA-OT). The detailed workflow of CINEMA-OT is as follows.

#### Rank initialization

To perform CINEMA-OT, we first need to initialize the expected matrix rank, representing the total signal number. We offer two possible approaches for rank initialization in CINEMA-OT.

Biwhitening^57^ is a recently developed method to remove independent heteroskedastic noise in data with inspirations from random matrix theory. It does diagonal matrix transformation of the data on both sides and thresholding based on the Marchenko–Pastur law^58^. After thresholding, we can get the true matrix rank and the matrix’s low-dimensional approximation. Mathematical details of biwhitening can be seen in ref. ^57^. In CINEMA-OT, we have implemented a version of biwhitening with fixed hyperparameters.

In large datasets, we suggest using prespecified rank values. Empirically, we have found that CINEMA-OT is robust to rank selection at certain ranges and can give a good performance when DimSize ∈ [20,50].

#### Signal selection with independent component analysis

Independent component analysis is already an established method in data analysis and has various implementations. Here we use the FastICA implementation from the package sklearn.decomposition^59^, with the ‘arbitrary-variance’ whitening scheme. Prior to FastICA, input data were PCA-transformed using Scanpy^60^.

To identify confounder signals and treatment-associated signals, we adopted a recently proposed cross rank coefficient^20^, which is able to quantify the functional dependence between ICA signals and query signals (in this case, the treatment signals). We use the implementation of this method from a modified faster version of the XICOR package in Python. The threshold of the cross rank coefficient is set to 0.05–0.75 in this study. Tuning the threshold parameter has a practical meaning in the algorithm. High thresholds correspond to less tolerance for false-positive treatment signals, which leads to local matching more similar to Mixscape analyses. Meanwhile, setting a low threshold means less tolerance for false-positive confounder signals and can lead to lower resolution of matching, which, in the extreme case, coincides with pseudo-bulk differential expression testing methods if the matching resolution is at cell-type level, and individual treatment effects are further aggregated.

#### Optimal transport matching

After selecting confounding signals, we perform matching across treatments via optimal transport, which provides a smooth transport map and does not require neighbor number selection. Here we consider the entropy-regularized optimal transport formulation, which can be efficiently solved by the Sinkhorn–Knopp algorithm^16^. In this formulation of the problem, the penalty coefficient acts as a hyperparameter influencing the resolution and smoothness of the transport map. We have empirically determined that the optimal value for the penalty coefficient often lies within the range (1 × 10^−6^ to 1 × 10^−3^) multiplied by the number of confounding signals.

**Algorithm 1** CINEMA-OT

**Require:** Count matrix PC embedding *X* ∈ *R*^*n* × *p*^, treatment vector $z\in {\left\{0,1\right\}}^{n}$, dimension size *r*, signal filtering threshold *d*, smoothness *s*.

1: $${{{\rm{DimSize}}}}\leftarrow r,{{{\rm{Thres}}}}\leftarrow d$$.

2: unmixing matrix *B*, source matrix *S* ← ICA(*X*, DimSize);

3: *c* ← zeros(DimSize)

4: **for** *i* = 1: DimSize **do**

5:  *c*_*i*_ ← xicor(*S*[: , *i*], *z*);      ⊳ Compute Chatterjee cross rank coefficient

6: **end for**

7: ${S}^{c}\leftarrow S[:,c < {{{\rm{Thres}}}}]$    ⊳ Thresholding to separate confounder signals *S*^*c*^

8: *M* ← OT(*S*^*c*^[*z* = 0], *S*^*c*^[*z* = 1], smoothness = s*S^*c*^. shape[1])   ⊳ M: Matching matrix

9: ITE ← *X*[*z* = 1]*M* − *X*[*z* = 0] ⊳ ITE matrix computation; can also be done for the original gene expression matrix

10: Downstream analysis.

**Algorithm 2** OT

**Require:** Confounder signals ${S}_{1}\in {{\mathbb{R}}}^{{n}_{1}\times p},{S}_{2}\in {{\mathbb{R}}}^{{n}_{2}\times p}$, weights *w*_1_ = None, *w*_2_ = None, smoothness *s*.

1: **if** *w*_1_ is None **then**

2:  *r* ← 1/*n*_1_, *c* ← 1/*n*_2_

3: **else**

4:  *r* ← *w*_1_/sum(*w*_1_), *c* ← *w*_2_/sum(*w*_2_)

5: **end if**

6: *D* ← PairwiseEuclideanDistance(*S*_1_, *S*_2_).

7: $A\leftarrow \exp (-D* D/s)$      ⊳ Elementwise multiplication for D here

8: $M={{{\rm{SinkhornKnopp}}}}(A,{{{\rm{setr}}}}=r,{{{\rm{setc}}}}=c)$     ⊳ Sinkhorn–Knopp algorithm

9: **return** *M*

### CINEMA-OT-W

In CINEMA-OT-W, the treated cells are first aligned by their 20 nearest neighbors in the untreated condition. Then Leiden clustering is performed on the full aligned cell set at a prespecified resolution (*r*). For each Leiden cluster, the cells from one of the conditions are subsampled such that the number of cells are the same for each condition in the same cluster. After subsampling, the confounder signals are independent of the treatment event. Therefore, the ICA procedure can be conducted on the subsampled data, and the confounder component selection is performed on the identified independent components. The entropic regularized OT can be later performed on the confounder components of either subsampled data or the full data. In the latter case, the ICA unmixing matrix computed from subsampled data is applied on the full data embedding. Notably, as control state indicates the cells in the normal states in most cases, we may assume that the untreated cells always cover the confounding states of the treated cells. In this case, the treatment effect of all treated cells can be computed by CINEMA-OT-W.

In practical datasets, the underlying confounders are often provided in terms of cell-wise labels (such as cell types), which indicates biological meaningful sampling labels. Therefore, apart from CINEMA-OT-W, CINEMA-OT offers users the ability to specify known confounder labels (for example cell type and cell cycle), without the need for a sampling procedure.

**Algorithm 3** CINEMA-OT-W

**Require:** Count matrix PC embedding *X* ∈ *R*^*n*×*p*^, treatment vector *z* ∈ {0, 1}^*n*^, dimension size *r*, signal filtering threshold *d*, smoothness *s*.

1: $${{{\rm{DimSize}}}}\leftarrow r,{{{\rm{Thres}}}}\leftarrow d,{X}_{0}\leftarrow X[z=0],{X}_{1}\leftarrow X[z=1]$$.

2: ${X}_{1}^{{\prime} }\leftarrow {{{{\rm{k}}}}-{{{\rm{NN}}}}}_{{X}_{0}}({X}_{1})$     ⊳ -NN: The average embedding of *k*-nearest neighbors

3: $${X}^{{\prime} }\leftarrow [{X}_{0};{X}_{1}^{{\prime} }],\,{X}_{new}\leftarrow {{{\rm{emptyList}}}}$$.

4: $$l\leftarrow {{{\rm{Leiden}}}}({X}^{{\prime} }),\,{z}_{new}\leftarrow {{{\rm{emptyList}}}}$$.

5: **for** $a=1:\max (l)$ **do**        ⊳ Cluster-wise sampling

6:   $$i\leftarrow {{{{\rm{argmax}}}}}_{i\in \{0,1\}}\{w[(z=i)\& (l=a)].{{{\rm{shape}}}}\}$$

7:   Append *X*[(*z* = 1 − *i*)*&*(*l* = *a*)] to *X*_new_

8:   Append $$(1-i) \times {{\bf{1}}}_{ X[(z=1-i) \& (l=a)]\left.\right).{{\mathrm{shape}}}[0]}$$ to *z*_new_

9:   Subsample *X*[(*z* = *i*)*&*(*l* = *a*)] to *X*[(*z* = 1 − *i*)*&*(*l* = *a*)]. shape[0] and append to *X*_new_

10:   Append $$\begin{array}{l}i \times {\bf{1}}_{\left.X\left[\left(z=1-i\right) \& \left(l=a\right)\right]\right).{\mathrm{shape}}\left[0\right]}\end{array}$$ to *z*_new_

11: **end for**

12: CINEMA-OT(*X*_new_, *z*_new_, *r*, *d*, *s*).

### Downstream analysis

#### Visualization and clustering of the ITE matrix

With the ITE matrix computed by matching counterfactuals, we are able to perform numerous standard analyses. We may employ dimensionality-reduction techniques, such as *t*-SNE, UMAP, or PHATE^61–63^, to visualize clusters in the response space. We may also employ clustering techniques, such as Leiden clustering^10^, to group cells by similarity of treatment responses.

#### Synergy analysis

For the synergy effect, we compare ITE matrices for two treatment conditions against the ITE matrix for the combined treatment. Formal derivation of the synergy score is given as follows.

Consider *D*_*A*=1,*B*=0_ as the ITE matrix for treatment *A* alone, *D*_*A*=0,*B*=1_ as the ITE matrix for treatment *B* alone, and *D*_*A*=1,*B*=1_ as the ITE matrix for the combined treatment. We may define a synergy matrix *Ψ* as:

$${{\varPsi }}={D}_{A = 1,B = 1}-\left({D}_{A = 1,B = 0}+{D}_{A = 0,B = 1}\right)$$

Where each entry *Ψ*_*g*,*c*_ represents the synergy score for gene *g* and cell *c*. To test whether a particular gene *g* has synergistic effect, we formulate the problem as if we should reject

$${H}_{0}:E({{{\varPsi }}}_{g,c})=0,\quad\forall c.$$

Here, if we apply only library-size-normalized data, we are aiming for additive synergy; if we further apply the log1p transformation, *H*_0_ would test for multiplicative synergy.

We assume that different cells are unlikely to have opposite synergy effects, allowing us to relax *H*_0_ as:

$${H}_{0}:E({\overline{{{\varPsi }}}}_{g,:})=0.$$

Assume the new *H*_0_ holds, then for each gene *g*, we compute the absolute value of empirical synergy as the synergy score:

$${{{\rm{Synergy\,score}}}}=| {\overline{{{\varPsi }}}}_{g,:}| .$$

In this case, identifying most synergistic genes among all genes can be turned into comparing the synergy score over all genes.

#### GSEA analysis

To assess differential gene expression significance, we used the non-parametric Wilcoxon signed-rank test. We used customized *P* value thresholds (1 × 10^−5^ in our study) and log-normalized expression difference thresholds (0.5 in our study) to identify significantly regulated genes. These genes are input into GSEApy for analysis by functional signatures^64,65^.

#### Attribution analysis

By clustering cells both by treatment responses (that is using the ITE matrix) and control condition clusters (that is cell subtypes), the matching matrix from CINEMA-OT can be coarse grained. The resulting coarse-grained matching matrix is of shape ResponseClusterNumber × ControlClusterNumber. Each column of the matrix gives the likelihood of a control condition cluster to have different modes of response. By reading each row of the matrix, we can attribute each response to the underlying control condition cluster.

Furthermore, to investigate the genes with confounder-specific treatment effect, we fit each gene’s normalized expression 𝑋 to the causal regression model, where 𝑧 denotes the treatment event, 𝑐 denotes the confounding factors and 𝛼, 𝛽, 𝛾 and constant are the linear regression coefficients and the intercept, respectively:

$$X=\alpha z+\beta c+\gamma cz+{\rm{constant}}$$

In this case, the confounder-specific effect part is *γcz*, whose significance can be established by classical linear regression theory. However, in our case, the noise term can stand for latent-factor-specific effect, thus not satisfying the assumption of classical regression. Therefore, here we instead quantify the *l*_2_-norm ratio between confounder-specific effect and the residual as an indicator of confounder-specific-effect significance.

### Data simulation and analysis

We used Scsim to simulate 1,000 gene by 5,000 cell-count matrices with 2–5 underlying cell states with 2 gene-regulation programs. For each cell state, we simulated a random discrete distribution to represent the corresponding response distribution of the cell state. Then the response count matrix of 500 genes × 5,000 cells was simulated and concatenated with the confounder count matrix.

For the Mixscape analysis, we implemented a simple version in Python that matches cells across conditions according to the descriptions in ref. ^11^. For Harmony-Mixscape analysis, we used the Python package harmonypy (https://github.com/slowkow/harmonypy) with default settings^38^, and applied Mixscape on the batch corrected embeddings returned by Harmony. For full OT analysis, we implemented a function that calls entropy-regularized optimal transport on the full PC embedding space with a tunable smoothness parameter. For scGen, CPA, ContrastiveVI and CellOT, the default model settings were used, consistent with those provided in their tutorials: https://scgen.readthedocs.io/en/stable/tutorials/scgen_perturbation_prediction.html (scGen); https://github.com/facebookresearch/CPA/blob/main/notebooks/demo.ipynb (CPA); https://colab.research.google.com/drive/1_R1YWQQUJzgQ6kz1XqglL5xZn8b8h1TX?usp=sharing (ContrastiveVI); https://github.com/bunnech/cellot/blob/main/configs/models/cellot.yaml (CellOT). For CellOT, we input principal component embeddings for training and evaluation.

To investigate the effect of hyperparameter settings on different methods, we performed parameter-sweep analysis for all tested methods. The sweeped hyperparameters for all methods are summarized as follows:

- Mixscape (the number of neighbors, *k*): [5, 10, 20 (Default), 50, 100];
- Harmony-Mixscape (*k*): [5, 10, 20 (Default), 50, 100];
- Full OT (regularization parameter *Ɛ*, smaller values resulted in instability): [0.1, 0.3, 1, 3];
- scGen (Kullback–Leibler (KL) divergence weight, *l*): [0, 5 × 10^–6^, 5 × 10^–5^ (Default), 5 × 10^–4^];
- CPA (adversary strength, *l*): [5, 20, 60 (Default), 200];
- ContrastiveVI (Wasserstein penalty, *l*_MMD_): [0 (Default),1 × 10^–4^, 1 × 10^–3^,1 × 10^–2^,1 × 10^–1^];
- CellOT (Frobenius norm regularization reg): [0.01, 0.1, 1 (Default), 10];
- CINEMA-OT (confounder threshold cutoff): [0.05, 0.1, 0.15, 0.2, 0.25]; (OT smoothness, *e*, based on the optimal cutoff): [1 × 10^–5^, 3 × 10^–5^, 1 × 10^–4^, 3 × 10^–4^, 1 × 10^–3^];
- CINEMA-OT-W (Leiden clustering resolution, r, based on optimal parameters of CINEMA-OT): [0.3, 0.6, 1, 1.2].

The parameter-sweep analysis results are listed in Supplementary Fig. 2. Based on the four metrics, the hyperparameter settings used throughout our benchmarking analysis were selected: *k* = 20 (Mixscape); k = 20 (Harmony-Mixscape); *Ɛ* = 0.1 (Full OT); *l* = 0 (scGen); *l* = 20 (CPA); *l*_MMD_ = 0 (ContrastiveVI); cutoff = 0.05, *e* = 1 × 10^–5^ (CINEMA-OT); *r* = 0.6 (CINEMA-OT-W).

On the basis of the optimized hyperparameters, the following analyses were performed:

1. The effect of differential abundance on single-cell treatment-effect analysis. To explore the effect of differential abundance on the performance of single-cell treatment-effect analysis methods, we selectively subsampled cells from half of the confounder clusters in the treated condition. The subsample ratio, which we refer to as differential abundance ratio, are selected as different levels: [1, 0.75, 0.5, 0.25, 0]. The case in which the DA ratio = 1 corresponds to no differential abundance effect, and when the DA ratio = 0, certain cell populations are not observed in the treated condition.
2. The effect of noise level on single-cell treatment effect analysis. To perform the analysis, the count matrix was subsampled according to the Scanpy function sc.pp.downsample_counts with the total_counts parameter specified to be (1, 0.5, 0.2, 0.1, 0.05) times the total count number of the original matrix.
3. Running time and peak memory usage. We conducted the scalability analysis by testing the run time and maximum memory usage of the different methods on subsampled interferon datasets, with cell numbers of 1,000, 2,000, 5,000, 10,000, 20,000, or 50,000. For Mixscape, scGen, CPA, and CINEMA-OT, the data were normalized and log-transformed, and we selected 773 highly variable genes using mean and dispersion thresholds provided by the default Scanpy function sc.pp.highly_variable _genes(adata, min_mean=0.0125, max_mean=3, min_disp=0.5). In the case of ContrastiveVI, which models the distribution of the count matrix, we used the original count matrix of highly variable genes.

### Benchmarking metrics

ASW, PCR, and ARI are batch-mixing and biological-preservation metrics used to evaluate batch-correction methods performance in the systematic benchmarking paper^41^. CINEMA-OT uses the first two metrics to evaluate mixing in confounder space, as a surrogate for correct matching that can still be measured when ground-truth labels are not present. ARI is used to evaluate the preservation of response clusters in ITE matrices estimated from simulated data. The PCR for ITE matrices is used to evaluate attribution accuracy of response, as in our experimental settings the response of each cell is conditionally independent of cell states conditioning on the response cluster assignments. For all metrics, we use the implementations from package scib^41^.

### Alzheimer’s scRNA-seq data

The Alzheimer’s scRNA-seq data were downloaded from https://drive.google.com/uc?id=1R1aN-LWUQ6c_N44n5-xjy2nEPzl7H0Dc. For Mixscape, scGen, CPA, and CINEMA-OT, the data were normalized and log-transformed. Two thousand highly variable genes were selected with the Seurat v3 approach implemented in Scanpy. As ContrastiveVI models the distribution of count matrix, the original count matrix of highly variable genes was used for ContrastiveVI. For CellOT, we input principal component embeddings computed from preprocessed highly variable genes for training and evaluation.

### Sci-Plex4 data

The Sci-Plex4 data were accessed from https://www.ncbi.nlm.nih.gov/geo/query/acc.cgi?acc=GSM4150379 with GEO accession number GSM4150379. The data are preprocessed via protocol https://github.com/manuyavuz/single-cell-analysis/blob/main/single_cell_analysis/datasets/sciplex.py. After preprocessing, we normalized and log-transformed the raw count matrix, then performed highly variable gene selection using mean and dispersion thresholds provided by the default Scanpy function sc.pp.highly _variable_genes(adata, min_mean=0.0125, max_mean=3, min_disp=0.5). Finally, we performed subsequent analysis, described in the main text,for Mixscape, scGen, CPA, and CINEMA-OT. The original count matrix of highly variable genes was used to evaluate ContrastiveVI. For CellOT, we input principal component embeddings computed from preprocessed highly variable genes for training and evaluation.

After estimating all metrics, each metric was rescaled so that the max value for all methods tested equals 1. Then we computed the average of batch mixing score (PCR) and label preservation score (the average of NMI and ARI) as the final metric used (Overall_score).

### Rhinovirus infection data

Primary human bronchial epithelial cells from healthy adult donors were obtained from commercial vendor (Lonza) and cultured at the air–liquid interface according to the manufacturer’s instructions (Stem Cell Technologies) using reduced hydrocortisone. Cells were kept at the air–liquid interface for 4 weeks before the experiment; maturation of beating cilia and mucus production was confirmed using a light microscope. Cells were then infected with mock or 1 × 10^5^ PFU human rhinovirus (HRV-01A, ATCC) per organoid, with or without exposure to 2% CSE. A single-cell suspension was collected by trypsin digestion at 5 d post-infection and submitted to scRNA-seq using The 10X Genomics single-cell 3^′^ protocol. The final dataset contains 26,420 cells in 4 samples (mock, RV, CSE, RVCSE). We performed normalization (by sc.pp.normalize_total), log1p transformation, hand selection of highly variable genes using mean and dispersion thresholds provided by the default Scanpy function sc.pp.highly_variable_genes(adata, min_mean=0.0125, max_mean=3, min_disp=0.5), scaled their values for PCA and Leiden clustering analysis. We annotated eight cell clusters on the basis of known cell-type markers of airway epithelial cells^66^: cycling basal, basal, hillock, secretory, pre-ciliated, ciliated, ionocyte, PNECs, and brush cells. CINEMA-OT analysis on mock–RV and mock–CSE was run with default parameters with smoothness = 1 × 10^−5^. Synergy analysis was performed with smoothness = 3 × 10^−5^.

### Interferon treatment data

#### PBMC processing and in vitro culture

The study was approved by Institutional Review Boards at Yale University (following Yale melanoma skin SPORE institutional review board protocol). Healthy donors consented to donation of peripheral blood for research use.

Human PBMCs were isolated using Lymphoprep density gradient medium (STEMCELL). PBMCs were plated at 1 million cells per ml and stimulated with 1,000 U ml^–1^ human IFN-α2 (R&D systems), 1,000 U ml^–1^ human IFN-β (PBL Assay Science 11415), 1,000 U ml^–1^ human IFN-γ (PBL Assay Science), 1 µg ml^–1^ human IFN-III/IL-29 (R&D Systems), 100 ng ml^–1^ human IL-6 (NCI Biological Resources Branch Preclinical Biologics Repository), 20 ng ml^–1^ human TNF (R&D Systems), and combinatorial cytokines IFN-β + IL-6, IFN-β + TNF, IFN-β + IFN-γ at indicated concentrations above for up to 48 h.

#### Cell enrichment and 10x sample preparation

Cultured cells were collected stained with TotalSeq anti-human hashtags C0251-C0260 (Clone LNH-94; 2M2, Biolegend, 1:1,000 dilution), viability dye (zombie red, Biolegend), and anti-human CD45-FITC (Clone HI30, Biolegend, 1:40 dilution) and enriched for live CD45^+^ cells using BD FACS Aria II. Sorted cells were then resuspended to 1,200 cells per µl and barcoded for multiplexed single-cell sequencing using 10x Genomics 5’v2 chemistry (10x Genomics, PN-1000263).

#### Sequencing and 10x sample alignment

Single-cell RNA sequencing libraries were sequenced on Illumina NovaSeq at read length of 150-bp pair-end and depth of 300 million reads per sample.

#### scRNA-seq data analysis

Data from three donors across Day 2 and Day 7 were concatenated together into labeled anndata objects for analysis. For each of the 6 samples, we filtered cells with fewer than 200 genes and we filtered genes expressed in fewer than 3 cells. For further quality control, cells with a high proportion of mitochondiral reads (>7%) were excluded. The distribution of genes per cell was visually inspected, and upper thresholds were selected on a per-sample basis to exclude doublets. For each of the samples, the upper threshold was selected as 6,000, 3,500, 4,000, 3,500, 4,500, or 3,500. Following filtering, the count data were normalized and log-transformed. Highly variable genes were selected using mean and dispersion thresholds provided by the default Scanpy function sc.pp.highly_variable_genes(adata, min_mean=0.0125, max_mean=3, min_disp=0.5). Highly variable genes were scaled for subsequent PCA and UMAP projection.

For individual treatment effect analysis, we additionally filtered T-cell-receptor genes, histocompatibility genes, and immunoglobulin genes from the highly variable gene set. Genes to be filtered were obtained from the HUGO database^67^. After filtering, highly variable genes were used for downstream visualization analysis.

CINEMA-OT analysis was run on each of the samples separately, with signal filtering threshold thres=0.5, smoothness=1e-4, and tolerance eps=1e-2, and preweights given by cell types. The implementation of other methods were consistent with the experiments conducted in the Sciplex dataset.

For the synergy analysis of donor 3 on day 2 (H3D2), we selected strongly synergistic genes by an absolute value threshold of 0.15.

### Reporting summary

Further information on research design is available in the Nature Portfolio Reporting Summary linked to this article.

## Online content

Any methods, additional references, Nature Portfolio reporting summaries, source data, extended data, supplementary information, acknowledgements, peer review information; details of author contributions and competing interests; and statements of data and code availability are available at 10.1038/s41592-023-02040-5.

### Supplementary information

Supplementary Information Supplementary Notes and Figs. 1–7.

Reporting Summary

## Data Availability

The Sciplex data were taken from the original publication^8^ (GSE139944) and the processed Alzheimer’s data were accessed from ContrastiveVI’s tutorial, with the original data from ref. ^40^ under GSE138852. The newly produced datasets (RV infection scRNA-seq data, combinatorial interferon stimulation scRNA-seq data) are available on Dryad^68^ in both formats of raw count files and preprocessed anndata files.

## Code availability

CINEMA-OT is implemented as a open-source Python package available at https://github.com/vandijklab/CINEMA-OT. An experimental version of CINEMA-OT that adopts OT solvers from the ott-jax library^39^ is available at https://github.com/theislab/pertpy.
