# Supplementary material for: Causal identification of single-cell experimental perturbation effects with CINEMA-OT
Source: Nat Methods. 2023 Nov 2;20(11):1769–79. doi: 10.1038/s41592-023-02040-5 (PMC10630139; doi:10.1038/s41592-023-02040-5)
Supplement: Supplementary file 1 — Supplementary Notes and Figs. 1–7. [file 41592_2023_2040_MOESM1_ESM.pdf]

nature methods

Article

https://doi.org/10.1038/s41592-023-02040-5

# Causal identification of single-cell experimental perturbation effects with CINEMA-OT

In the format provided by the
authors and unedited

## Contents

**1 Supplementary notes 1** **2**

**2 Supplementary notes 2** **4**

**3 Supplementary figures** **7**

## List of Figures

1 An example of distance distortion with noisy confounder signals . . . . . . . . . . . 3

1 CINEMA-OT still identifies correct confounder in data with moderate differential abundance but fails in data with significant differential abundance . . . . . . . . . 7

2 Parameter sweep analysis for different single-cell level treatment effect analysis methods . . . . . . . . . . . . . . . . . . . . . . . . . . . . . . . . . . . . . . . 7

3 CINEMA-OT validation on the Rhinovirus infection data estimating the causal effect of CSE . . . . . . . . . . . . . . . . . . . . . . . . . . . . . . . . . . . . . 8

4 CINEMA-OT validation on the Rhinovirus infection data estimating the causal effect of RV . . . . . . . . . . . . . . . . . . . . . . . . . . . . . . . . . . . . . . 9

5 UMAP visualizations of batch-wise CINEMA-OT counterfactual space . . . . . . 10

6 Comparison of different different cell types' chemokine response with stacked violin plots of gene expression . . . . . . . . . . . . . . . . . . . . . . . . . . . . . . . 11

# 1 Supplementary notes 1

Here we give an rigorous treatment of the causal framework and underlying assumptions in CINEMA-OT.

**Assumption 1 (Formal): Independent sources and noise.** *Confounding factors and treatment events* $(s_1, ..., s_l, z)$ *are independent random variables. The treatment event* $z \in \{-1, 1\}$ *with* $P(z = 1) = p$*.*

**Assumption 2 (Formal): Linearity of source signal combinations.** *The expression of each gene can be linearly decomposed as the mixing of noisy confounding signals and treatment-associated signals. Without loss of generality, we assume the concatenated random vector* $(s_1 + e_1, ..., s_l + e_l, z + e_z)$ *is whitened and at most only one of the* $(s_1 + e_1, ..., s_l + e_l, z + e_z)$ *is Gaussian. The observed data matrix* $X \in \mathbb{R}^{n \times d}$ *is generated by mixing of i.i.d sampled noisy signals plus i.i.d noise terms* $\epsilon$*.*

In our formulation, to be more realistic, we consider both the biological variation terms $e$ and the measurement noise $\epsilon$. For simplicity, we may understand the signal $s$ as cell types and $e$ as biological variations contributing to the heterogeneity within each cell type. Given assumption 1 and 2, denote the gene count matrix as $X \in \mathbb{R}^{n \times d}$, and it is generated by a random vector $\boldsymbol{x} \in \mathbb{R}^m$ with the mixing matrix $A$. Then the data generation mechanism is given by

$$\boldsymbol{x}^i \overset{\text{i.i.d}}{\sim} \begin{bmatrix} s_1 + e_1 \\ \vdots \\ s_l + e_l \\ z + e_z \end{bmatrix}; \quad \boldsymbol{\epsilon}^i \text{ i.i.d}; \quad X = [\boldsymbol{x}^1, \boldsymbol{x}^2, ..., \boldsymbol{x}^n]^T A + [\boldsymbol{\epsilon}^1, \boldsymbol{\epsilon}^2, ..., \boldsymbol{\epsilon}^n]^T. \tag{1}$$

As $n \to \infty$, by the subspace consistency established in [1], we have the first $l+1$ identified principal components of form:

$$\hat{\boldsymbol{x}} \overset{\text{i.i.d}}{\sim} B \begin{bmatrix} s_1 + e_1 \\ \vdots \\ s_l + e_l \\ z + e_z \end{bmatrix}. \tag{2}$$

Here $B$ represents a linear transform. Note after PCA preprocessing, different components $s_i + e_i$ or $z + e_z$ are still independent and at most only one of the factors is Gaussian. As a result, the ICA identifiability theorem [2] can be directly applied on $\boldsymbol{x}$ to unmix the independent components, which means the confounding factors are identifiable, up to a permutation:

$$W^{\text{ICA}} \hat{\boldsymbol{x}} \overset{\text{i.i.d}}{\sim} \begin{bmatrix} s_1 + e_1 \\ \vdots \\ s_l + e_l \\ z + e_z \end{bmatrix}, \text{ up to a permutation.} \tag{3}$$

Finally, a statistical test on the difference between untreated and treated distributions for each independent component can be performed to distinguish the confounding factors from the treatment event signal.

Our theoretical justification here reveals that: 1. The (noisy version of) confounder terms are identifiable with the ICA transform up to a permutation; 2. The contributions of noise terms are not distorted as the relative ratio between $s, z$ and $e$ are preserved in the output. These two points supports the validity of using CINEMA-OT for matching according to the identified (noisy) confounders.

Moreover, we are able to show that in the same setting, a non-linear neural network based architecture named conditional (variational) autoencoder (used as the key component in scGen [3], compositional autoencoder [4], and contrastiveVI [5] along with other tools) can fail to identify the confounder signals.

In the model of conditional (variational) autoencoders, the latent space can be represented as two parts $\boldsymbol{s} = [\boldsymbol{s}_0; z]$. First is the basal embedding $\boldsymbol{s}_0$, where the embedding of cell distributions from

different conditions overlap; The other is the treatment signal $z$, which can be transformed into a treatment associated embedding. The generative model can be written as the following form:

$$\boldsymbol{x} \sim \int P_\theta(\boldsymbol{x}|\boldsymbol{s}_0, z)P(\boldsymbol{s})d\boldsymbol{s} \tag{4}$$

Here the setting of Gaussian $\boldsymbol{s}$ corresponds to a variational autoencoder design and a deterministic $\boldsymbol{s}$ conditioning on $\boldsymbol{x}$ corresponds to the vanilla autoencoder design, and $\theta$ denotes the decoder network parameters. In our context, we suppose the conditional (variational) autoencoder is optimized by the reconstruction loss with respect to $\hat{\boldsymbol{x}}$ in eq (2) with / without Gaussian constraint:

$$(\hat{\theta}, \hat{\boldsymbol{s}}) = \text{argmin}_{\theta,\boldsymbol{s}}||F_\theta(\boldsymbol{s}_0, z) - \hat{\boldsymbol{x}}|| \tag{5}$$

Even we assume the function above is perfectly optimized, and the learned $\boldsymbol{s}_0$ is indeed independent of $z$, $\boldsymbol{s}_0$ can be still an arbitrary transform of the noisy confounder signals. This is based on the following fundamental result from [6, 7]:

**Theorem.** *[6, 7] Let $\boldsymbol{x}$ be a d-dimensional random vector of any distribution. Then there exists a transformation $F : \mathbb{R}^d \to \mathbb{R}^d$ such that the components of $\boldsymbol{x}' := F(\boldsymbol{x})$ are independent, and each component has a standardized Gaussian distribution. In particular, $\boldsymbol{x}'_1$ equals a monotonic transformation of $\boldsymbol{x}_1$.*

The above theorem means, for any arbitrary injective transformation $F([s_1 + e_1, ..., s_l + e_l]^T)$, its first component can be used as the first independent component in $\boldsymbol{s}_0$. This immediately leads to two observations: 1. The confounder terms are no longer identifiable up to a permutation; 2. Even though the information of confounder can be preserved up to a injective transformation, the distance measure on the latent space can be dramatically distorted. To see why the second point holds, suppose $s_1$ is a binary r.v. with $P(s_1 = 1) = P(s_1 = -1) = 0.5$, and $\frac{Var(s_1)}{Var(e_1)}$ is a sufficiently large constant. We can see in this case the distance based on $s_1 + e_1$ is almost determined by $s_1$; however, the distance measure after an injective transform can be almost determined by $e_1$ (Supplementary Note Figure 1). In summary, our theoretical analysis suggests that in the model setting, the classical ICA can give consistent distance measures based on confounder space, while the non-linear conditional autoencoder approaches suffer from non-identifiability and distance distortion, leading to less meaningful latent spaces.

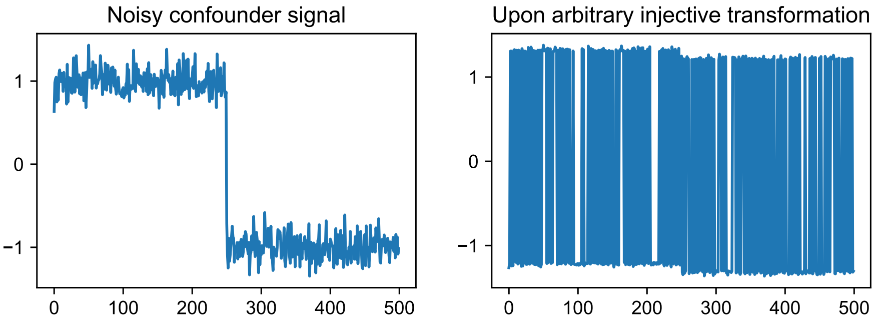


Supplementary Note Figure 1: An example of distance distortion with noisy confounder signals.

Empirically, we also observe our method can successfully reveal confounding variations even with non-linear interactions between confounding factors and treatment events. In this case, the causal matching may be performed according to a non-one-to-one transform of the full confounder signal, which is not consistent with the full confounder distribution but still meaningful in preserving a part of confounder information. More specifically, it interpolates between a single-cell level causal matching and a cluster/population-level causal matching.

Finally, given the identified confounding factors, the problem of treatment effect estimation can be solved by standard potential outcome framework. The framework has mainly four assumptions, which are discussed in detail in causal inference textbooks [8]:

1. Stable Unit Treatment Assumption (SUTVA): Samples are independent without interference; 2. Ignorability: there are no unmeasured confounders; 3. Consistency; 4. Positivity: for a given confounder, the probability of perturbation is neither 0 or 1.

## 2 Supplementary notes 2

There are a number of existing methods that perform perturbation effect analysis in single-cell omics data. Several pioneering works in the field propose the use of factor models to identify perturbation effects, including LRICA [9], MIMOSCA [10], scMAGeCK [11], MUSIC [12], and WGCNA/STM [13]. More recently, Mixscape [14] estimates single-cell level perturbation effects by matching between neighboring cells in the shared k-NN graph across conditions. Additionally, several deep learning-based frameworks learn perturbation responses from scRNA-seq data via autoencoders as deep factor models, including scGen [3], compositional autoencoder (CPA) [4], and ContrastiveVI [5]. Finally, CellOT [15] proposes a neural network that learns a non-linear transport map aligning cells from different treatment conditions.

Despite these efforts, none of the existing methods achieve guaranteed confounder identification, which leads to interpretable causal effect estimation by aligning cells with the same confounder states across conditions. Among the alternative single-cell treatment effect analysis methods, Mixscape and CellOT do not model the confounding variation. Moreover, Mixscape considers the nearest neighbor relationship on the entire gene expression space instead of distributional matching, which may lead to vulnerability to cell outliers and unbalanced mixing. While the auto-encoder based methods model confounder variation in general, they can suffer from the fundamental limitation of un-identifiability [7] (See Supplementary notes for a detailed explanation), which can reduce their power in identifying ground truth confounding variations. CINEMA-OT is the first method that achieves confounder identification as well as distributional matching for the task of single-cell treatment effect analysis.

Independent component analysis (ICA) has found widespread use in the field of causal inference. One of the most established methods among these is LiNGAM [16], which infers the directed causal relationship between features, with the directions derived by combining the independent noise assumption and ICA identifiability. The LiNGAM framework has been applied to a number of tasks, including: causal discovery for time-series data [17, 18], identifying the features most responsible for an intervention [19], and causal learning across multiple groups [20]. The key distinction between these methods and CINEMA-OT is that the LiNGAM-based methods solve a causal discovery task at the feature (gene) level, while CINEMA-OT seeks to identify the causal effect of an intervention on individual observations (cells). Therefore, LiNGAM-based methods are not appropriate for our task but may find applications in gene regulatory network inference [21–23] or the causal discovery of spatially-regulated gene networks in spatial omics data [24–26].

## References


[1] Dan Shen, Haipeng Shen, and J Marron. A general framework for consistency of principal component analysis. *Journal of Machine Learning Research*, 2016.

[2] Pierre Comon. Independent component analysis, a new concept? *Signal processing*, 36(3): 287–314, 1994.

[3] Mohammad Lotfollahi, F Alexander Wolf, and Fabian J Theis. scgen predicts single-cell perturbation responses. *Nature methods*, 16(8):715–721, 2019.

[4] Mohammad Lotfollahi, Anna Klimovskaia Susmelj, Carlo De Donno, Yuge Ji, Ignacio L Ibarra, F Alexander Wolf, Nafissa Yakubova, Fabian J Theis, and David Lopez-Paz. Compositional perturbation autoencoder for single-cell response modeling. *BioRxiv*, 2021.

[5] Ethan Weinberger, Chris Lin, and Su-In Lee. Isolating salient variations of interest in single-cell transcriptomic data with contrastivevi. *bioRxiv*, 2021.

[6] Aapo Hyvärinen and Petteri Pajunen. Nonlinear independent component analysis: Existence and uniqueness results. *Neural networks*, 12(3):429–439, 1999.

[7] Ilyes Khemakhem, Diederik Kingma, Ricardo Monti, and Aapo Hyvarinen. Variational autoencoders and nonlinear ica: A unifying framework. In *International Conference on Artificial Intelligence and Statistics*, pages 2207–2217. PMLR, 2020.

[8] Guido W Imbens and Donald B Rubin. *Causal inference in statistics, social, and biomedical sciences*. Cambridge University Press, 2015.

[9] Atray Dixit, Oren Parnas, Biyu Li, Jenny Chen, Charles P Fulco, Livnat Jerby-Arnon, Nemanja D Marjanovic, Danielle Dionne, Tyler Burks, Raktima Raychowdhury, et al. Perturb-seq: dissecting molecular circuits with scalable single-cell rna profiling of pooled genetic screens. *cell*, 167(7):1853–1866, 2016.

[10] Britt Adamson, Thomas M Norman, Marco Jost, Min Y Cho, James K Nuñez, Yuwen Chen, Jacqueline E Villalta, Luke A Gilbert, Max A Horlbeck, Marco Y Hein, et al. A multiplexed single-cell crispr screening platform enables systematic dissection of the unfolded protein response. *Cell*, 167(7):1867–1882, 2016.

[11] Lin Yang, Yuqing Zhu, Hua Yu, Xiaolong Cheng, Sitong Chen, Yulan Chu, He Huang, Jin Zhang, and Wei Li. scmageck links genotypes with multiple phenotypes in single-cell crispr screens. *Genome biology*, 21(1):1–14, 2020.

[12] Bin Duan, Chi Zhou, Chengyu Zhu, Yifei Yu, Gaoyang Li, Shihua Zhang, Chao Zhang, Xiangyun Ye, Hanhui Ma, Shen Qu, et al. Model-based understanding of single-cell crispr screening. *Nature communications*, 10(1):1–11, 2019.

[13] Xin Jin, Sean K Simmons, Amy Guo, Ashwin S Shetty, Michelle Ko, Lan Nguyen, Vahbiz Jokhi, Elise Robinson, Paul Oyler, Nathan Curry, et al. In vivo perturb-seq reveals neuronal and glial abnormalities associated with autism risk genes. *Science*, 370(6520):eaaz6063, 2020.

[14] Efthymia Papalexi, Eleni P Mimitou, Andrew W Butler, Samantha Foster, Bernadette Bracken, William M Mauck, Hans-Hermann Wessels, Yuhan Hao, Bertrand Z Yeung, Peter Smibert, et al. Characterizing the molecular regulation of inhibitory immune checkpoints with multimodal single-cell screens. *Nature genetics*, 53(3):322–331, 2021.

[15] Charlotte Bunne, Stefan G Stark, Gabriele Gut, Jacobo Sarabia del Castillo, Kjong-Van Lehmann, Lucas Pelkmans, Andreas Krause, and Gunnar Ratsch. Learning single-cell perturbation responses using neural optimal transport. *bioRxiv*, 2021.

[16] Shohei Shimizu, Patrik O Hoyer, Aapo Hyvärinen, Antti Kerminen, and Michael Jordan. A linear non-gaussian acyclic model for causal discovery. *Journal of Machine Learning Research*, 7(10), 2006.

[17] Aapo Hyvärinen, Kun Zhang, Shohei Shimizu, and Patrik O Hoyer. Estimation of a structural vector autoregression model using non-gaussianity. *Journal of Machine Learning Research*, 11 (5), 2010.

[18] Hongxia Chen. Ica based causality inference between variables. In *2017 IEEE 17th International Conference on Communication Technology (ICCT)*, pages 1906–1910. IEEE, 2017.

[19] Patrick Blöbaum and Shohei Shimizu. Estimation of interventional effects of features on prediction. In *2017 IEEE 27th International Workshop on Machine Learning for Signal Processing (MLSP)*, pages 1–6. IEEE, 2017.

[20] Shohei Shimizu. Joint estimation of linear non-gaussian acyclic models. *Neurocomputing*, 81: 104–107, 2012.

[21] Sara Aibar, Carmen Bravo González-Blas, Thomas Moerman, Vân Anh Huynh-Thu, Hana Imrichova, Gert Hulselmans, Florian Rambow, Jean-Christophe Marine, Pierre Geurts, Jan Aerts, et al. Scenic: single-cell regulatory network inference and clustering. *Nature methods*, 14(11):1083–1086, 2017.

[22] Carmen Bravo González-Blas, Seppe De Winter, Gert Hulselmans, Nikolai Hecker, Irina Matetovici, Valerie Christiaens, Suresh Poovathingal, Jasper Wouters, Sara Aibar, and Stein Aerts. Scenic+: single-cell multiomic inference of enhancers and gene regulatory networks. *bioRxiv*, pages 2022–08, 2022.

[23] Kenji Kamimoto, Blerta Stringa, Christy M Hoffmann, Kunal Jindal, Lilianna Solnica-Krezel, and Samantha A Morris. Dissecting cell identity via network inference and in silico gene perturbation. *Nature*, pages 1–10, 2023.

[24] Livnat Jerby-Arnon and Aviv Regev. Dialogue maps multicellular programs in tissue from single-cell or spatial transcriptomics data. *Nature biotechnology*, 40(10):1467–1477, 2022.

[25] David S Fischer, Anna C Schaar, and Fabian J Theis. Modeling intercellular communication in tissues using spatial graphs of cells. *Nature Biotechnology*, pages 1–5, 2022.

[26] Mingze Dong and Yuval Kluger. GEASS: Neural causal feature selection for high-dimensional biological data. In *International Conference on Learning Representations*, 2023.

# 3 Supplementary figures

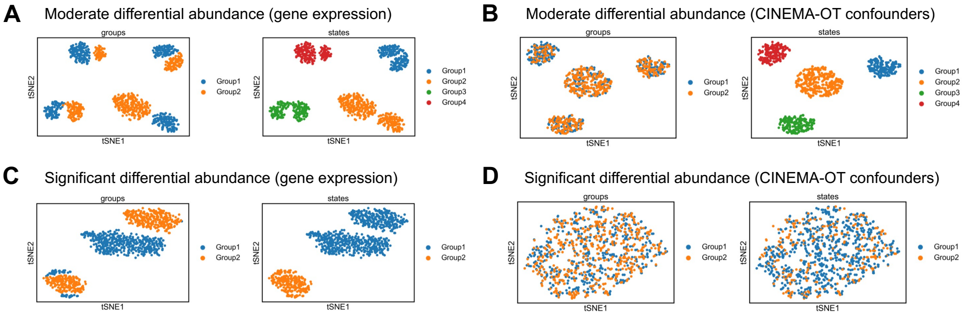


Supplementary Figure 1: CINEMA-OT method still identifies correct confounder in data with moderate differential abundance but fails in data with significant differential abundance. **A.** UMAP projection of original gene expression for the synthetic dataset with moderate differential abundance, colored by the treatment condition and cell type. **B.** UMAP projection of CINEMA-OT confounder space, colored by the treatment condition and cell type. **C.** UMAP projection of original gene expression for the synthetic dataset with significant differential abundance, colored by the treatment condition and cell type. **D.** UMAP projection of CINEMA-OT confounder space, colored by the treatment condition and cell type.

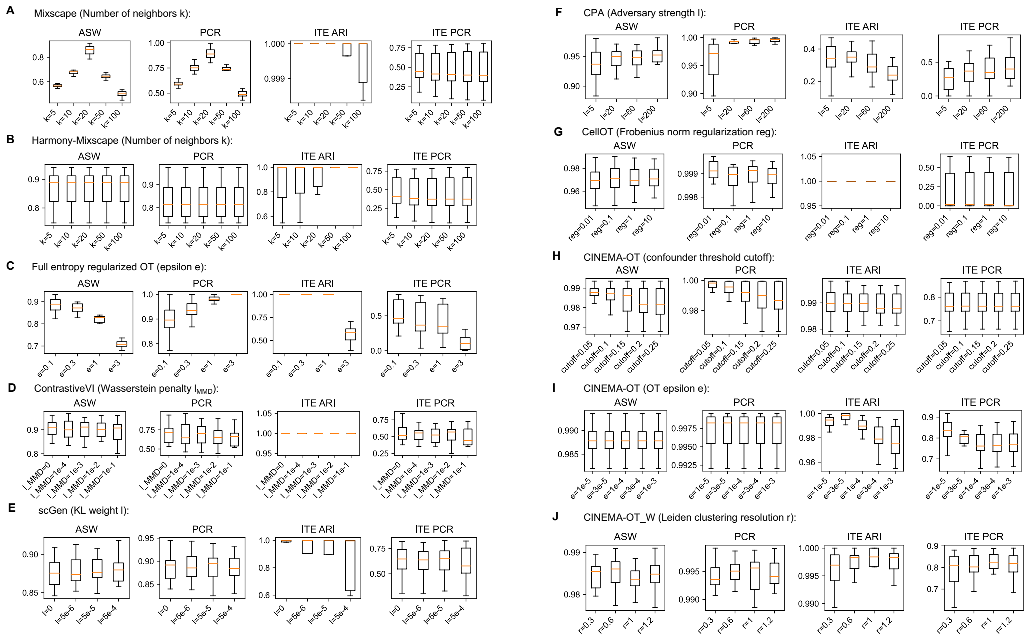


Supplementary Figure 2: Parameter sweep analysis for different single-cell level treatment effect analysis methods using boxplot (n=15 for confounder embedding metrics, n=12 for ITE metrics). The top/lower hinge represents the upper/lower quartile and whiskers extend from the hinge to the largest/smallest value no further than 1.5 $\times$ interquartile range from the hinge, respectively. The median is used as the center.

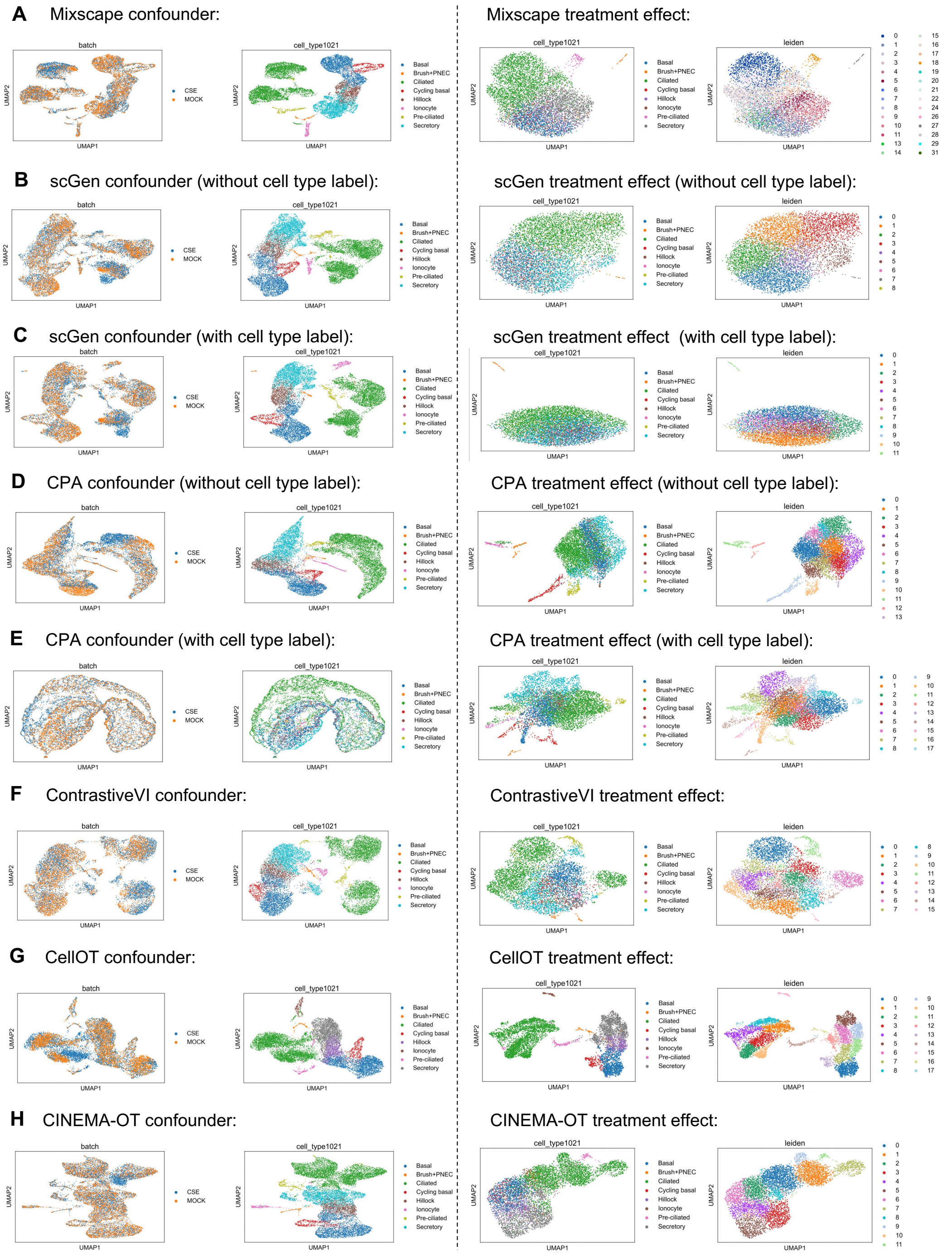


Supplementary Figure 3: CINEMA-OT validation on the Rhinovirus infection data estimating the causal effect of CSE. **A-H.** Different methods' confounder space visualization (colored by treatment condition and cell types) and treatment effect visualization (colored by Leiden clusters and cell types).

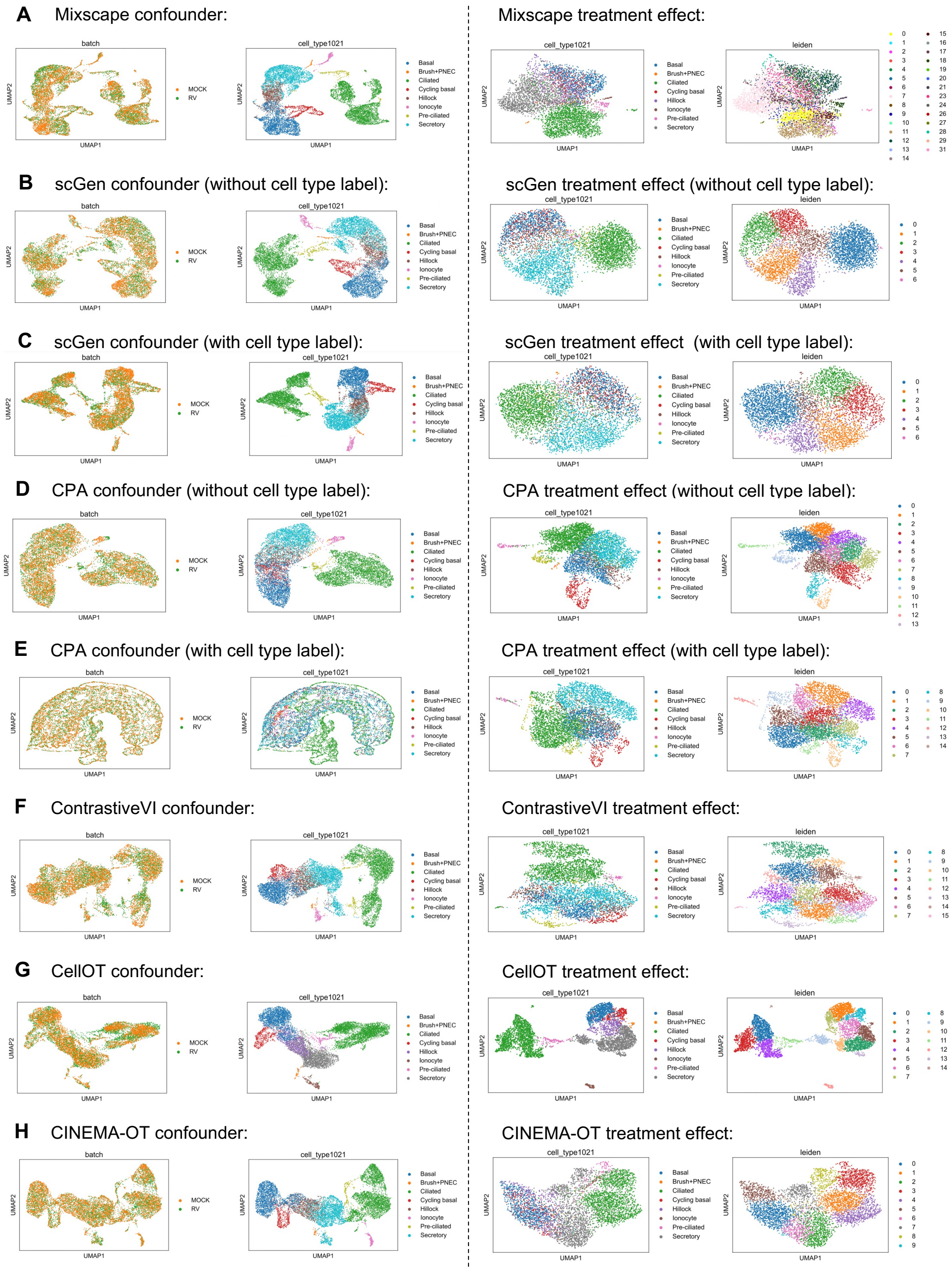


Supplementary Figure 4: CINEMA-OT validation on the Rhinovirus infection data estimating the causal effect of RV. **A-H.** Different methods' confounder space visualization (colored by treatment condition and cell types) and treatment effect visualization (colored by Leiden clusters and cell types).

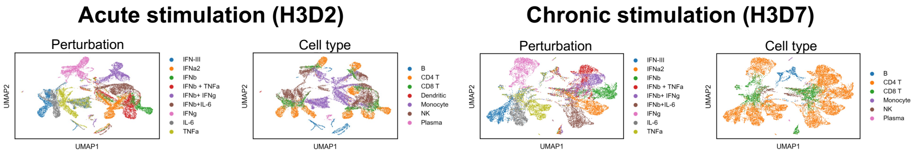


Supplementary Figure 5: UMAP visualizations of batch-wise CINEMA-OT counterfactual space, colored by perturbation and cell type.

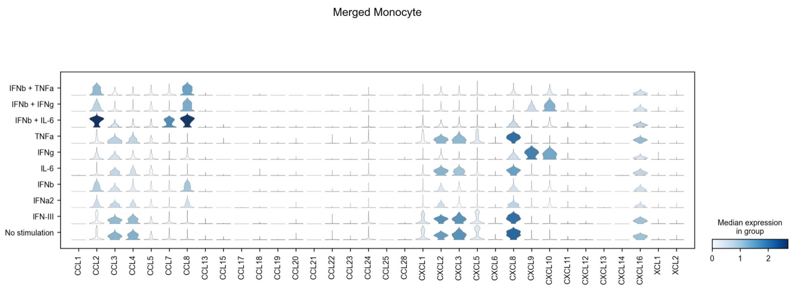


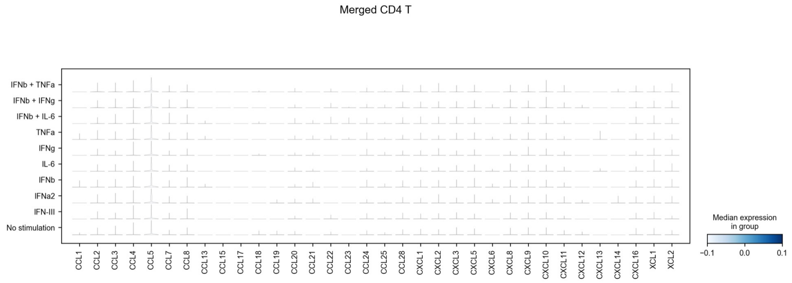


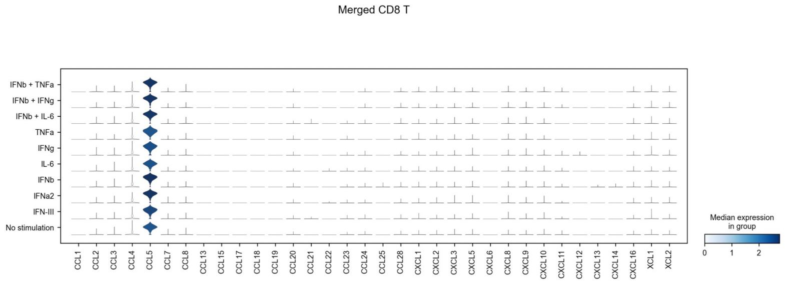


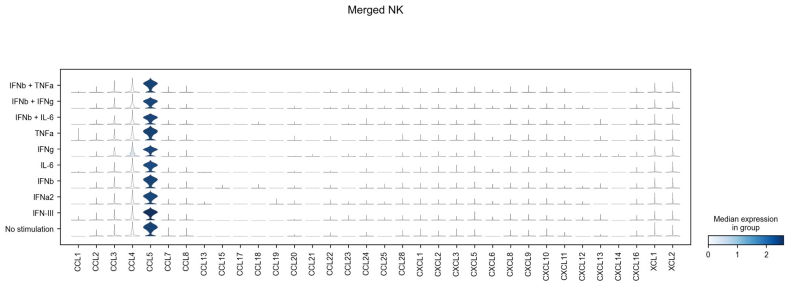


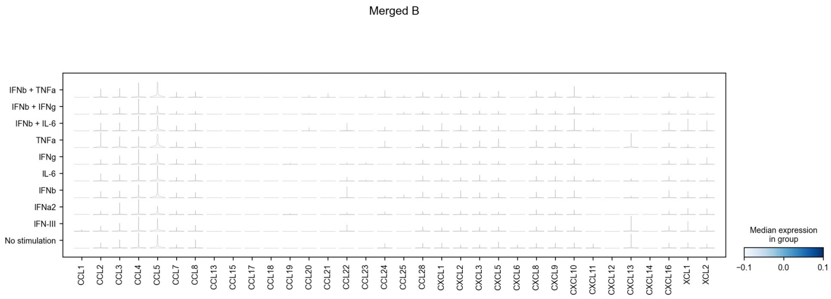


Supplementary Figure 6: Comparison of different different cell types' chemokine response with stacked violin plots of gene expression. Systematic differential expressions of chemokines across interferon perturbations are only observed in monocytes.
