# Supplementary material for: Causal identification of single-cell experimental perturbation effects with CINEMA-OT
Source: Nat Methods. 2023 Nov 2;20(11):1769–79. doi: 10.1038/s41592-023-02040-5 (PMC10630139; doi:10.1038/s41592-023-02040-5)
Supplement: Supplementary file 2 — Reporting Summary [file 41592_2023_2040_MOESM2_ESM.pdf]

nature portfolio

Corresponding author(s): Ellen F. Foxman, Jeffrey J. Ishizuka, Rahul M. Dhodapkar, David van Dijk

Last updated by author(s): Jul 19, 2023

Reporting Summary

Nature Portfolio wishes to improve the reproducibility of the work that we publish. This form provides structure for consistency and transparency in reporting. For further information on Nature Portfolio policies, see our Editorial Policies and the Editorial Policy Checklist.

Statistics

For all statistical analyses, confirm that the following items are present in the figure legend, table legend, main text, or Methods section.

n/a	Confirmed	
☐	☒	The exact sample size (*n*) for each experimental group/condition, given as a discrete number and unit of measurement
☐	☒	A statement on whether measurements were taken from distinct samples or whether the same sample was measured repeatedly
☐	☒	The statistical test(s) used AND whether they are one- or two-sided<br>*Only common tests should be described solely by name; describe more complex techniques in the Methods section.*
☐	☒	A description of all covariates tested
☐	☒	A description of any assumptions or corrections, such as tests of normality and adjustment for multiple comparisons
☐	☒	A full description of the statistical parameters including central tendency (e.g. means) or other basic estimates (e.g. regression coefficient) AND variation (e.g. standard deviation) or associated estimates of uncertainty (e.g. confidence intervals)
☒	☐	For null hypothesis testing, the test statistic (e.g. *F*, *t*, *r*) with confidence intervals, effect sizes, degrees of freedom and *P* value noted<br>*Give P values as exact values whenever suitable.*
☒	☐	For Bayesian analysis, information on the choice of priors and Markov chain Monte Carlo settings
☒	☐	For hierarchical and complex designs, identification of the appropriate level for tests and full reporting of outcomes
☒	☐	Estimates of effect sizes (e.g. Cohen's *d*, Pearson's *r*), indicating how they were calculated

Our web collection on statistics for biologists contains articles on many of the points above.

Software and code

Policy information about availability of computer code

Data collection	The Cell Ranger pipeline (v3.0.2 for rhinovirus infection experiment / v6.1.2 for combinatorial cytokine stimulation experiment) was used to generate the count matrices from the newly generated datasets.
Data analysis	Our main analysis uses the CINEMA-OT Python package (v0.0.3), available at https://github.com/vandijklab/CINEMA-OT; The sciplex data is preprocessed with additional code available at https://github.com/manuyavuz/single-cell-analysis/blob/main/single_cell_analysis/datasets/sciplex.py (Commit id: 44e31959bca1618b05f897a524c4f4ce42d5b8dd). Analyses were performed using Python 3.9. Other relevant software and versions: scanpy (v1.9.1), anndata (v0.8.0), umap (v0.5.3), numpy (v1.22.3), scipy (v1.8.1), pandas (v1.5.2), scikit-learn (v1.1.1), statsmodels (v0.13.2), python-igraph (v0.9.10), louvain (v0.7.1), pynndescent (v0.5.7), scSim (Commit id: 20011651341c70cbda8e41f6446380b4435693ab), harmonypy (v0.0.5), CPA (Commit id: f16b3dcdd59ef1b7f863de9c6623a47a25c24dee), contrastivevi (v0.1.0), scGen (v2.1.0), cellot (Commit id: df112863fb7b22a7a94ea57d404a7f57ae3bddd9), gseapy (v0.10.8).

For manuscripts utilizing custom algorithms or software that are central to the research but not yet described in published literature, software must be made available to editors and reviewers. We strongly encourage code deposition in a community repository (e.g. GitHub). See the Nature Portfolio guidelines for submitting code & software for further information.

Data

Policy information about availability of data

All manuscripts must include a data availability statement. This statement should provide the following information, where applicable:

- Accession codes, unique identifiers, or web links for publicly available datasets
- A description of any restrictions on data availability
- For clinical datasets or third party data, please ensure that the statement adheres to our policy

The data of Sciplex is taken from the original publication (GSE139944) and the processed Alzheimer data is accessed from ContrastiveVI's tutorial, with the original data under the accession number GSE138852. The newly produced datasets (Rhinovirus infection scRNA-seq data, combinatorial interferon stimulation scRNA-seq data) are available on Dryad (https://doi.org/10.5061/dryad.4xgxd25g1) in both formats of raw count files and preprocessed anndata files.

Human research participants

Policy information about studies involving human research participants and Sex and Gender in Research.

Reporting on sex and gender	Sex and gender analysis is not considered in our study.
Population characteristics	For the single cell sequencing of rhinovirus infection, the donor was a female between the ages of 30-40. For the interferon stimulation experiment, all donors are healthy at the time of peripheral blood donation. All donors were between the ages of 20-40. Two donors were male and one donor was female.
Recruitment	For the rhinovirus infection experiment, The de-identified primary human airway epithelial cells used in this paper were obtained commercially from Lonza. For the interferon stimulation experiment, healthy donors consented to donation of peripheral blood for research use in accordance with Yale IRB #2000033353. Healthy donors were recruited on a voluntary basis by advertisement local to the Yale Cancer Center. Self-selection bias cannot be excluded, nor can bias arising from the limited recruitment pool.
Ethics oversight	For the rhinovirus infection experiment, Lonza guarantees that all tissue utilized for human cell products is ethically obtained with donor informed consent in accordance with processes approved by an Institutional Review Board or comparable independent review body. The interferon stimulation study was approved by Institutional Review Boards at Yale University (following Yale melanoma skin SPORE IRB protocol).

Note that full information on the approval of the study protocol must also be provided in the manuscript.

Field-specific reporting

Please select the one below that is the best fit for your research. If you are not sure, read the appropriate sections before making your selection.

☒ Life sciences ☐ Behavioural & social sciences ☐ Ecological, evolutionary & environmental sciences

For a reference copy of the document with all sections, see nature.com/documents/nr-reporting-summary-flat.pdf

Life sciences study design

All studies must disclose on these points even when the disclosure is negative.

Sample size	For the airway epithelial cell infection, the final dataset has one biological replicate, and contains 26420 cells in 4 samples from each condition (mock, RV, CSE, RVCSE). For the interferon stimulation experiment, we sample PBMC from 3 healthy donors, containing 103518 cells after preprocessing and filtering. No statistical method was used to predetermine sample size. The primary outcome of the studies was verification of the capabilities of the computational method, not verification of the biological significance of relevant findings. A sample size of three was sufficient for demonstration of the method.
Data exclusions	No dataset is excluded. For the interferon stimulation experiment, for each of the 6 samples, we filtered cells with less than 200 genes and we filtered genes expressed in fewer than 3 cells. For further quality control, cells with a high proportion of mitochondrial reads (> 7\%) were excluded. The distribution of genes per cell was visually inspected and upper thresholds selected on a per-sample basis to exclude doublets. For each of the samples, the upper threshold was selected as [6000,3500,4000,3500,4500,3500] respectively.
Replication	For the airway epithelial cell infection, the scRNA-seq experiement was performed on 1 set of replicate experiments. For the interferon stimulation experiment, the results from the three healthy donors should be regarded as replicates. There was homogeneity among the three healthy donors with regard to interferon stimulation response, and therefore all attempts at replication were successful.
Randomization	For both experiments, no randomization procedure was performed. For the rhinovirus infection experiment, there is only one replicate. For the interferon stimulation experiment, each healthy donor’s samples were subjected to all experimental conditions for comparison, and

therefore there is no role for randomization.

Blinding

For the interferon stimulation experiment, each healthy donor's samples were subjected to all experimental conditions for comparison, therefore blinding among healthy donor identity was not necessary. All experimental conditions were pooled for processing and genomic analysis, therefore blinding of experimental condition was not necessary. Additionally, for both datasets, transcriptional state changes in cell culture would not be evaluable by simple observation, so blinding among experimental conditions is not necessary.

Reporting for specific materials, systems and methods

We require information from authors about some types of materials, experimental systems and methods used in many studies. Here, indicate whether each material, system or method listed is relevant to your study. If you are not sure if a list item applies to your research, read the appropriate section before selecting a response.

Materials & experimental systems

n/a	Involved in the study
☐	☒ Antibodies
☒	☐ Eukaryotic cell lines
☒	☐ Palaeontology and archaeology
☒	☐ Animals and other organisms
☒	☐ Clinical data
☒	☐ Dual use research of concern

Methods

n/a	Involved in the study
☒	☐ ChIP-seq
☒	☐ Flow cytometry
☒	☐ MRI-based neuroimaging

Antibodies

Antibodies used

No antibodies are involved in the airway epithelial infection experiment. For the interferon stimulation experiment: TotalSeq anti-human hashtags C0251-C0260 (Clone LNH-94; 2M2, Biolegend, 1:1000 dilution); Anti-human CD45-FITC (Clone HI30, Biolegend, 1:40 dilution)

TotalSeq anti-human hashtag C0251 (Clone LNH-94; 2M2) (Cat:394661) (Lot:B343252)
TotalSeq anti-human hashtag C0252 (Clone LNH-94; 2M2) (Cat:394663) (Lot:B337758)
TotalSeq anti-human hashtag C0253 (Clone LNH-94; 2M2) (Cat:394665) (Lot:B342838)
TotalSeq anti-human hashtag C0254 (Clone LNH-94; 2M2) (Cat:394667) (Lot:B346859)
TotalSeq anti-human hashtag C0255 (Clone LNH-94; 2M2) (Cat:394669) (Lot:B338441)
TotalSeq anti-human hashtag C0256 (Clone LNH-94; 2M2) (Cat:394671) (Lot:B342026)
TotalSeq anti-human hashtag C0257 (Clone LNH-94; 2M2) (Cat:394673) (Lot:B341069)
TotalSeq anti-human hashtag C0258 (Clone LNH-94; 2M2) (Cat:394675) (Lot:B339940)
TotalSeq anti-human hashtag C0259 (Clone LNH-94; 2M2) (Cat:394677) (Lot:B334825)
TotalSeq anti-human hashtag C0260 (Clone LNH-94; 2M2) (Cat:394679) (Lot:B338860)
Anti-human CD45-FITC (Clone HI30) (Cat: 304038) (Lot:B348058)

Validation

Links to manufacturer site for each antibody used are provided, technical data sheets and lot specific certificate of analysis that confirm species reactivity and application are available for each antibody.

TotalSeq anti-human hashtag C0251 (Clone LNH-94; 2M2) (Cat:394661) (Lot:B343252)
https://www.biolegend.com/en-ie/products/totalseq-c0251-anti-human-hashtag-1-antibody-17162

TotalSeq anti-human hashtag C0252 (Clone LNH-94; 2M2) (Cat:394663) (Lot:B337758)
https://www.biolegend.com/en-ie/products/totalseq-c0252-anti-human-hashtag-2-antibody-17163

TotalSeq anti-human hashtag C0253 (Clone LNH-94; 2M2) (Cat:394665) (Lot:B342838)
https://www.biolegend.com/en-ie/products/totalseq-c0253-anti-human-hashtag-3-antibody-17164

TotalSeq anti-human hashtag C0254 (Clone LNH-94; 2M2) (Cat:394667) (Lot:B346859)
https://www.biolegend.com/en-ie/products/totalseq-c0254-anti-human-hashtag-4-antibody-17165

TotalSeq anti-human hashtag C0255 (Clone LNH-94; 2M2) (Cat:394669) (Lot:B338441)
https://www.biolegend.com/en-ie/products/totalseq-c0255-anti-human-hashtag-5-antibody-17166

TotalSeq anti-human hashtag C0256 (Clone LNH-94; 2M2) (Cat:394671) (Lot:B342026)
https://www.biolegend.com/en-ie/products/totalseq-c0256-anti-human-hashtag-6-antibody-18373

TotalSeq anti-human hashtag C0257 (Clone LNH-94; 2M2) (Cat:394673) (Lot:B341069)
https://www.biolegend.com/en-ie/products/totalseq-c0257-anti-human-hashtag-7-antibody-18374

TotalSeq anti-human hashtag C0258 (Clone LNH-94; 2M2) (Cat:394675) (Lot:B339940)
https://www.biolegend.com/en-ie/products/totalseq-c0258-anti-human-hashtag-8-antibody-18375

TotalSeq anti-human hashtag C0259 (Clone LNH-94; 2M2) (Cat:394677) (Lot:B334825)
https://www.biolegend.com/en-ie/products/totalseq-c0259-anti-human-hashtag-9-antibody-18376

TotalSeq anti-human hashtag C0260 (Clone LNH-94; 2M2) (Cat:394679) (Lot:B338860)
https://www.biolegend.com/en-ie/products/totalseq-c0260-anti-human-hashtag-10-antibody-18433

Anti-human CD45-FITC (Clone HI30) (Cat:304038) (Lot:B348058)
https://www.biolegend.com/de-at/sean-tuckers-tests/fitc-anti-human-cd45-antibody-707
